# A Proteomic Approach to Analyze the Aspirin-mediated Lysine Acetylome*[Fn FN2]

**DOI:** 10.1074/mcp.O116.065219

**Published:** 2016-12-05

**Authors:** Michael H. Tatham, Christian Cole, Paul Scullion, Ross Wilkie, Nicholas J. Westwood, Lesley A. Stark, Ronald T. Hay

**Affiliations:** From the ‡Centre for Gene Regulation and Expression, Sir James Black Centre, School of Life Sciences, University of Dundee, Dow Street, Dundee, DD1 5EH. UK;; §Computational Biology, School of Life Sciences, University of Dundee, Dow Street, Dundee, DD1 5EH. UK;; ¶Biological Chemistry and Drug Discovery, School of Life Sciences, University of Dundee, Dow Street, Dundee, DD1 5EH. UK;; ‖School of Chemistry and Biomedical Sciences Research Complex, University of St Andrews and EaStCHEM, North Haugh, St Andrews, Fife. KY16 9ST. UK;; **Edinburgh Cancer Research Centre, Institute of Genetics and Molecular Medicine, University of Edinburgh, EH4 2XU UK

## Abstract

Aspirin, or acetylsalicylic acid is widely used to control pain, inflammation and fever. Important to this function is its ability to irreversibly acetylate cyclooxygenases at active site serines. Aspirin has the potential to acetylate other amino acid side-chains, leading to the possibility that aspirin-mediated lysine acetylation could explain some of its as-yet unexplained drug actions or side-effects. Using isotopically labeled aspirin-d_3_, in combination with acetylated lysine purification and LC-MS/MS, we identified over 12000 sites of lysine acetylation from cultured human cells. Although aspirin amplifies endogenous acetylation signals at the majority of detectable endogenous sites, cells tolerate aspirin mediated acetylation very well unless cellular deacetylases are inhibited. Although most endogenous acetylations are amplified by orders of magnitude, lysine acetylation site occupancies remain very low even after high doses of aspirin. This work shows that while aspirin has enormous potential to alter protein function, in the majority of cases aspirin-mediated acetylations do not accumulate to levels likely to elicit biological effects. These findings are consistent with an emerging model for cellular acetylation whereby stoichiometry correlates with biological relevance, and deacetylases act to minimize the biological consequences of nonspecific chemical acetylations.

Aspirin, also known as acetylsalicylic acid (ASA)^1^ is the most widely used drug in the world (1) and is taken to treat acute pain, fever and inflammation. It also has long term applications in the prophylactic treatment of heart attacks, strokes, and pathological blood clot formation (2). An emerging role for aspirin is in the prevention of some malignant transformations, such as colorectal cancer (3–6). Aspirin administration can be associated with various undesirable side-effects including gastrointestinal bleeding, ulcerations, nephrotoxicity and tinnitus.

Aspirin is a non-steroidal anti-inflammatory drug (NSAID), and is the only NSAID known to function by irreversible modification of the cyclooxygenases COX-1 and COX-2. Acetylation at active site serines 530 and 516 respectively, inhibits prostaglandin and thromboxane synthesis (7, 8). Aspirin has also been shown to acetylate the ε-amino-group of lysine side-chains in cellular and extracellular proteins including serum albumin (9), fibrinogen (10), hemoglobin (11), p53 (12) and glucose-6-phosphate dehydrogenase (13, 14). Work using radiolabeled aspirin (15), and acetylated lysine (AcK)-specific antibodies (16) has shown that aspirin can acetylate cellular and extracellular proteins. Considering the salience of reversible enzymatic protein acetylation (17), these observations lend weight to the hypothesis that aspirin-mediated lysine acetylation may explain some of the currently unexplained functions of the drug (16).

To date, proteomic approaches to identify sites of protein acetylation by aspirin have either lacked site-level data (14, 18), or used chemically modified forms of aspirin with unknown consequences on drug action (19). Critically, the extent of acetylation has not been investigated even at the single protein level, and so aspirin's true potential to interfere with cellular systems via acetylation is still unclear.

We have developed a method that employs a highly specific peptide enrichment strategy in combination with isotopically labeled aspirin-d_3_ that does not alter its chemical reactivity. This allows unambiguous, proteome-wide analysis of aspirin-mediated lysine acetylation in any biological context. We identified over 12,000 AcK-d_3_ sites in 3763 proteins from HeLa cells, and show that most detectable endogenous acetylations, with exception of histone N-terminal tails, are greatly enhanced by aspirin. However, this huge up-regulation of cellular acetylation still only influences a very small proportion of any particular protein, as site occupancies are below 1% for the vast majority of acetylations. We found that aspirin-mediated acetylations are mainly opposed by the action of endogenous deacetylases, and inhibition of HDAC6 enhances aspirin acetylations and increases aspirin-mediated cytotoxicity. These findings show that the endogenous deacetylase system is capable of blunting aspirin's acetylation potential and highlight the considerable task involved in pinpointing acetylations that may explain currently obscure modes of aspirin action.

## EXPERIMENTAL PROCEDURES

### 

#### 

##### Cell Survival Assays

Approximately 20,000 HeLa cells per well were seeded in a 96-well, white, flat-bottomed tissue-culture plate (Sigma, UK) in a volume of 100 μl culture medium (DMEM lacking phenol red, (Thermo Fisher Scientific, UK) supplemented with 2 mm glutamine and 10% fetal calf serum, plus penicillin/streptomycin). Cells were incubated for 18 h at 37 °C at 5% CO_2_. Dilutions of either aspirin or salicylic acid were made in culture medium to final concentrations of 20 mm, 10 mm, 5 mm, 2 mm, 1 mm, and 0.5 mm. A zero drug dilution was made containing only DMSO at the same concentration as in the dilutions (aspirin and SA were dissolved and stored in DMSO). To begin exposure to salicylate cell culture medium was replaced with the salicylate dilutions in quadruplicate. Cells were cultured at 37 °C and 5% CO_2_ for 6, 24, or 48 h. To assess cell viability 100 μl ATP assay buffer (50 mm Tris/phosphate pH 7.8, 16 mm MgCl_2,_ 2 mm DTT, 2% v/v Triton-X-100, 30% v/v (37.8% w/v) glycerol, 1% w/v BSA, 0.25 mm d-luciferin, 8 μm sodium pyrophosphate tetra-basic decahydrate, 500 ng/ml Luciferase) was added to each well, before sealing with clear film and agitating at 900 rpm and 20 °C for 10 min. Luminescence was measured using an EnVision Multilabel plate reader (Perkin Elmer, UK). Readings were normalized to the zero-drug control for each set of replicates. In experiments using co-treatment with KDAC inhibitors, bufexamac was used at 0.25 mm or nicotinamide at 20 mm, cells were exposed for 24 h, and salicylate dilutions of 20 mm, 10 mm, 5 mm, 2.5 mm, 1.25 mm, and 0.625 mm were used.

##### Synthesis of Aspirin-d_3_

2.18 g salicylic acid (Sigma Aldrich) was mixed with 5g acetic anhydride-d_6_ (Sigma Aldrich) in an Erlenmeyer flask before addition of 8 drops (∼400 μl) of 85% orthophosphoric acid. The solution was mixed by agitation and warmed to 70 °C in a water bath for 15 min. Unreacted acetic anhydride-d_6_ was destroyed by addition of 14 drops (∼700 μl) cold ultra-pure water. Crystallization was initiated by addition of 14.5 ml ultra-pure water and transfer of the solution to an ice-bath for 30 min. Crystals of aspirin-d_3_ were removed from solution by filtration onto filter paper under suction on a Buckner funnel. Crystals were washed with ∼20 ml ice-cold ultra-pure water, then dried by 15 min suction. Recrystallization of Aspirin-d_3_ was then performed: The crystals were dissolved in 10 ml ethanol while gently warming. Recrystallization was triggered by addition of 27 ml warm ultra-pure water, followed by slow cooling at ambient temperature, then rapid cooling on ice. Crystals were filtered out of solution onto filter paper using a Buchner funnel and suction for 15 min, then dried on a fresh sheet of filter paper in a glass beaker loosely covered with tissue-paper for 2 days. Unlabeled Aspirin was synthesized by an identical method.

##### NMR Analysis of Aspirin

^1^H and ^13^C spectra were obtained with a Bruker Avance II 400 MHz, Bruker Avance 500 MHz or a Bruker Avance III 500 MHz spectrometer with the solvent peak used as the internal standard. NMR spectra were processed using TopSpin 3.1 (PC version) or MestReNova.

##### MS Analysis of Aspirin

Saturated solutions of commercially sourced aspirin, in house synthesized aspirin, and in house synthesized aspirin-d_3_ were made in acetonitrile. 1:1000 dilutions in acetonitrile of each was analyzed on a QExactive mass spectrometer in negative mode using a direct infusion flow rate of 20 μl·min^−1^, linked to a HESI-II probe in Ion Max API source (Thermo Scientific) running at 4 kV, 300 °C and S-lens RF level of 50. Settings for full MS scans were; scan range = 172–185 *m*/*z*, resolution = 70000, AGC target = 1e6, maximum injection time = 20ms. Spectra were acquired over 25 scans and viewed in Xcalibur Qual Browser.

##### Kinetic Analysis of Aspirin and Salicylic Acid in Cultured Cells and Cell Medium

HeLa cells were cultured in six well plates (9.5 cm^2^ area per well) in DMEM supplemented with FCS to 10% such that final cell counts at the end of the time-course were 1 to 1.5 million. Cells were exposed to 5 mm aspirin (unlabeled) for 0.25, 0.5, 1, 2, 6, 16, or 24 h by replacement of 2 ml medium. To assess concentrations of aspirin (ASA) and salicylic acid (SA) in both culture medium and cells the following method was used for each well of cultured cells: 1 ml culture medium was removed and snap-frozen before storage at −80 °C. The remaining medium was removed and the cells washed three times with 1 ml ice-cold PBS. Cells were lysed by addition of 100 μl lysis buffer (20 mm Tris pH 7.5, 150 mm NaCl, 1% triton-X100, plus Roche protease inhibitor mixture), and incubated on ice with orbital shaking for ∼10 min. Lysates were removed from wells and snap frozen before storage at −80 °C. This process was repeated in triplicate (*n* = 3) for each time point. For each time point a fourth well of cells was grown in parallel to allow estimation of cell numbers and cell volume using an automated cell counter (Countess, Invitrogen, UK). These values were used to calculate total cell volume, which was between 2 and 3 μl depending on the time point.

ASA and SA were measured in medium and lysate samples using UV absorption with in-line UPLC (Acquity) running a BEH-C18 50 × 2.1 mm 1.7 μm particle column. A 3 min gradient protocol was used running from 2 to 95% acetonitrile (in 0.1% formic acid) then back to 2%. For our system SA presented a λ_max_ = 304 nm and retention time (RT) 1.49 min and ASA a λ_max_ = 276 nm at RT 1.42 min. Calibration curves were defined over a range of ASA and SA concentrations from 0.1 to 1000 μg/ml. Sample concentrations of ASA and SA were calculated by reference to these calibration curves.

Measured data were modeled using Copasi (v4.16 - build 104) (20). Data were fit to a simple model for the diffusion of aspirin and salicylic acid between medium and cells and for the hydrolysis of aspirin in both compartments as shown in supplemental Fig. S1*A*. The five species shown in supplemental Fig. S1*A*, and two compartments were defined; Medium (2 ml) and cells (2 μl). Experimental data were weighted manually toward the more confident measurements from the culture medium (weight 1.0), and away from the cellular measurements (weight 0.2) due to both the inherent errors of the lower concentration measurements (actual concentration measurements in the cell extracts were in the order of 10 times lower than for the medium measurements) and the fact that these values included the extra variable of cell counting and size measurements. The seven rate constants shown in supplemental Fig. S1*A* were modeled along with the starting concentrations of SA and ASA in the medium. All other starting concentrations were fixed at zero. The in and out rate constants for ASA were constrained to be equal, as were those for SA. Repeated iterations of parameter estimation resulted in the fits shown in supplemental Fig. S1*B*, and the final rate constant values shown in supplemental Fig. S1*C*. Parameter scanning was used to predict the effect of the starting concentration of aspirin in the medium on the maximum cellular concentration of aspirin (supplemental Fig. S1*E*).

##### Antibodies

Anti-acetylated lysine antibody (250 μg.ml^−1^) (ImmuneChem, Burnaby, Canada) was used at 1:2000 dilutions 16 h 4 °C in immunoblotting experiments. Anti-Rabbit HRP (Sigma) was used as secondary antibody at 1:2000 dilutions for 1 h at room temperature.

##### Purification of Acetylated Peptides from Cultured Human Cells

Twenty-one 150 mm diameter plates were used to culture HeLa cells to ∼70% confluency in 20 ml DMEM, 10% FCS. In batches of seven plates, the cells were exposed for 6 h with DMSO only, aspirin in DMSO or aspirin-d_3_ in DMSO, to a final DMSO concentration of 0.13% and aspirin concentration of 5 mm. Cells were washed three times with PBS and for each set of seven plates, the resultant cell pellet was lysed in four pellet volumes of 6 m urea, 2 m thiourea in 100 mm Tris/HCl pH 8.5 (lysis buffer). The three lysates were sonicated on ice (Branson sonifier, narrow tip, 40%) for a total sonication time of 140 s with 20 s on, 20 s off cycles. Protein yields were determined by Bradford's assay to be 40–45 mg per lysate. Samples were reduced by addition of DTT to 1 mm for 30 min at room temperature, followed by alkylation with 5 mm iodoacetamide during centrifugation at 20,000 × *g* for 30 min at room temperature in the dark. Any remaining debris was cleared from supernatants by 0.2 μm filtration. Per condition, 40 mg protein was carried forward. Each was digested by incubation with 1:200 (w:w) ratio LysC/protein (200 μg, Wako, Japan) at room temperature for 4 h. Peptide samples were diluted four times with 50 mm ammonium bicarbonate before digestion each with 1:400 (w:w) ratio trypsin/protein (100 μg - SIGMA trypsin gold) for 16 h at room temperature. Digestions were halted by acidification with addition of 10% trifluoroacetic acid (TFA) solution to pH ∼2–3 (to ∼0.6% TFA v:v). Precipitate was removed by centrifugation at 3000 × *g* for 15 mins before 0.2 μm filtration. For each sample peptides were purified by C18 reverse phase chromatography using spin columns (two Waters Sep-Pak, 6cc, 1 g cartridges per condition) as described by the manufacturers. Peptides were eluted from columns by 70% ACN in 0.1% TFA. Peptide concentrations were estimated using OD 260 and OD 280 measurements and the Warburg-Christian method. A volume equivalent to 300 μg peptide was removed for each batch (for use as “Crude” analysis) and these along with the remaining peptide samples were lyophilized in a vacuum centrifuge attempting to avoid over-drying. The 300 μg Crude samples were each resuspended to a concentration of 0.85 mg.ml^−1^ in 0.5% acetic acid, 0.1% TFA and carried forward for MS analysis. The remaining peptides were resuspended in 2 ml IP buffer (50 mm Tris/HCl pH 8.0, 100 mm NaCl). Any undissolved peptides were removed by centrifugation at 20000g for 30 min. Peptide solutions were requantified by the Warburg-Christian method to be 3.9–4.2 mg. ml^−1^. To purify acetylated lysine peptides, immune affinity chromatography was used. Briefly: 60μl anti-acetylated lysine agarose beads (ImmuneChem) pre-equilibrated with IP buffer was mixed with each peptide solution for 16 h at 4 °C. The resin was washed three times with 1 ml IP buffer before elution of peptides with three washes with 100 μl 0.1% TFA. Peptide solutions were desalted using two 4 ply STAGE tips per prep, and lyophilized peptide elutions resuspended in 60 μl 0.5% acetic acid, 0.1% TFA. These were carried forward for MS analysis as 'IP' samples for each treatment.

For the SILAC experiment investigating dynamics of aspirin-mediated lysine acetylation a single 150 mm dish of cells was cultured for each time point, except the 8h aspirin, 0h recovery condition, which had 8 plates because it was being used as a reference (see Fig. 6 C for experimental design). All treatments and purifications were carried out essentially as described above but scaling down to account for lower starting protein amounts. Five SILAC mixes labeled A to E were prepared, each with initial total protein amounts of 2–5 mg, and with the three SILAC conditions being mixed 1:1:1 (protein w:w:w) according to Bradford's assay.

##### MS Analysis of Peptide Samples

Peptide samples were analyzed by LC-MS/MS on a Q Exactive mass spectrometer (Thermo Fisher Scientific) coupled to an EASY-nLC 1000 liquid chromatography system (Thermo Scientific) via an EASY-Spray ion source (Thermo Fisher Scientific). Peptides were fractionated on a 75 μm × 500 mm EASY-Spray column (Thermo Scientific) over various gradient lengths from 90 min to 240 min. The following describes the typical analytical set-up, but further specific details of MS run conditions can be found within the raw data files. Precursor ion full scan spectra were acquired over (*m*/*z* 300 to 1,800) with a resolution of 70,000 at *m*/*z* 200 (target value of 1,000,000 ions, maximum injection time 20 ms). Up to ten data dependent MS^2^ spectra were acquired with a resolution of 17,500 at *m*/*z* 200 (target value of 500,000 ions, maximum injection time 60 ms). Ions with unassigned charge state, and singly or highly (>8) charged ions were rejected. Intensity threshold was set to 2.1 × 10^4^ units. Peptide match was set to preferred, and dynamic exclusion option was enabled (exclusion duration 40 s). The mass spectrometry proteomics raw data files have been deposited to the ProteomeXchange Consortium via the PRIDE partner repository (http://www.ebi.ac.uk/pride/archive/) with the dataset identifier PXD003530 for the label-free site ID analysis, and PXD004995 for the occupancy and SILAC half-life analysis.

##### MS Data Analysis

Raw MS data files were processed using MaxQuant software (version 1.3.0.5) (21, 22) and searched against UniProtKB human proteome (86749 sequences - 13/06/2012) and the built-in “contaminants” list. The variable modification for lysine acetylated by aspirin was defined in Andromeda to allow automated database searching for Acetyl-d_3_ K. Specificity was considered only for lysines, composition was set to H_-1_C_2_OHx_3_ (monoisotopic mass 45.029394924), position at peptide C termini was excluded, and two diagnostic peaks were defined; H_8_C_7_ONHx_3_ (128.1028942181) and H_11_C_7_ON_2_Hx_3_ (145.1294433196). For RAW data analysis in MaxQuant enzyme specificity was set to trypsin/P, cleaving C-terminal to lysine or arginine. Lysine and arginine were selected as special amino acid and a maximum number of three missed cleavages were allowed. Carbamidomethylation of cysteines was set as a fixed modification and oxidation of methionines, acetylation of protein N termini, acetylation of lysines and d_3_-acetylation of lysines were set as variable modifications. A minimum peptide length was set to seven amino acids and a maximum peptide mass was 5000 Da. A false discovery rate of 1% was set as a threshold at protein, peptide and site levels, and a mass deviation of 6 ppm was set for main search and 20 ppm for MS2 peaks. MaxQuant output files have been deposited to the ProteomeXchange Consortium via the PRIDE partner repository (see above), and MS/MS spectra can be viewed on MS-Viewer (http://msviewer.ucsf.edu/prospector/cgi-bin/msform.cgi?form=msviewer) using the search keys 4iznb5zvfu (label-free study) and kjfmg24iwf (SILAC study).

The final list of aspirin-mediated lysine acetylation sites was created by filtering the MaxQuant output removing sites by the following criteria; (a) Peptides derived from the decoy database, (b) Sites with localization probability lower than 0.75, (c) Peptides with mass error greater than 2 ppm after mass recalibration. (d) Peptides of only bovine origin. (e) Peptides that had the best evidence for modification come from a sample not treated with aspirin-d_3_. Protein copy number values were calculated using the label-free proteomic ruler method (23) using a ploidy value of 3.4 based on the 76–80 chromosome estimate for HeLa cells, compared with the usual 46 for human cells.

##### Sequence Logo Analysis

Sequence logos were generated by plogo v1.2.0 (24). For aspirin sites 12069 31 residue sequence windows were submitted, of which 11370 were processed. For the endogenous sites 1473 were submitted, of which 1273 were processed. “Endogenous” sites were defined as those sites where the file containing the spectrum with the best localization probability was found in nonaspirin-treated cells. Background was the human proteome, *n* = 660373.

##### Secondary structure and solvent exposure predictions

All peptides were expanded to their whole protein “parent” and submitted to Jpred v4 (25) for protein secondary structure prediction. Jpred has length and time limits for making predictions where any protein longer than 800 amino acids or shorter than 20 amino acids, or where a prediction takes longer than 1 h will not get a prediction. Thus, a small number of proteins do not get a secondary structure prediction. DisEMBL disorder prediction (26) was performed via JabaWS (27). The central lysine residue for each peptide was then interrogated in its parent protein for its secondary structure and disorder prediction and assigned as being either in helical, beta strand or coil, and disordered or non-disordered region. Any peptides missing secondary structure predictions were ignored. The counts for each of the five states were summed across all peptides in a particular set and then compared between sets. As predicted secondary structure from Jpred can only be one of three states the comparison was at the level of proportion of each state between sets. The Wald method was used to determine the 95% confidence interval on the proportions of each state in each data set:

$$\begin{array}{c} S\prime =S+\frac{1.96^{2}}{2}\\ n\prime =n+1.96^{2}\\ p\prime =\frac{S\prime }{n\prime }\\ CI_{0.95}=1.96\times \sqrt{\frac{p\prime \left(1-p\prime \right)}{n\prime }} \end{array}$$

Where *S* is the number of lysines in a given state, and *n* is the total number of lysines in a given dataset. Similar methods were applied to solvent exposure calculations, which is also provided by Jpred. In this case the majority of lysines are predicted to be solvent exposed so comparisons were made on those with a predicted solvent exposure of 25% or less.

##### SILAC Analysis of Aspirin-mediated Acetylation Decay

MS data derived from the five SILAC mixtures described above were processed by MaxQuant essentially as described above for the label-free experiment. The match between runs, and requantify options were selected in MaxQuant to allow identifications among “experiments” to be used in all, and to provide ratios for modified peptides even in absence of a heavy counterpart. The reported list of identified sites was filtered to remove: (a) Peptides derived from the decoy database, (b) sites with localization probability lower than 0.75, (c) peptides with mass error greater than 2 ppm after mass recalibration, (d) peptides with no reported SILAC ratios. This removed 2061 sites. SILAC ratios were manually normalized using the median normalization factor created by MaxQuant for the Crude data, for each reported ratio. These normalized SILAC ratios for each “Mix” were used to calculate relative acetylation at each time point. Acetylation was set to 100% for time *t* = 8 h exposure to aspirin-d_3_ (0 h recovery), and % acetylated for all other time points was calculated by average of two methods using all three SILAC ratios reported in a single mix. For example, for “Mix D” (*L* = 8 h Aspirin, *M* = 8 h aspirin + 0.25 h recovery, and *H* = 8 h aspirin + 0.5 h recovery);

% acetylated at 8.25 h according to M/L ratio;

(Eq. 1) $$A_{8.25}^{1}=100{\left(M/L\right)}_{mixD}$$

or % acetylated at 8.25 h according to H/L and H/M ratios;

(Eq. 2) $$A_{8.25}^{2}=100{\left(H/L\right)}_{mixD}/{\left(H/M\right)}_{mixD}$$

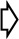
 % acetylated at 8.25 h,

(Eq. 3) $$A_{8.25}=\left(A_{8.25}^{1}+A_{8.25}^{2}\right)/2$$

% acetylated at 8.5 h according to H/L ratio;

(Eq. 4) $$A_{8.5}^{1}=100{\left(H/L\right)}_{mixD}$$

or % acetylated at 8.5 h according to M/L and H/M ratios;

(Eq. 5) $$A_{8.5}^{2}=100{\left(M/L\right)}_{mixD}\cdot {\left(H/M\right)}_{mixD}$$

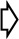
 % acetylated at 8.5 h;

(Eq. 6) $$A_{8.5}=\left(A_{8.5}^{1}+A_{8.5}^{2}\right)/2$$

Data points corresponding to mixes without SILAC ratios were not included in the analysis. Data were fit to a single phase exponential decay by non-linear regression using GraphPad Prism version 6.0f (GraphPad Software, Inc.). Plateau was constrained to 0% acetylation and only positive K values were considered. Any sites reported by Prism as “Too few points,” “Hit constraint,” or “Not converged” were rejected. For the remainder, only sites with R squared value of 0.85 and a minimum of 9 data points were shortlisted. Any of these with acetylation profiles characteristic of exogenous proteins (extremely short half-lives and potentially of bovine origin), were rejected. Of a total of 6942 sites remaining after initial filtering, this secondary filtering left a list of 1480 sites of aspirin-mediated acetylation of human proteins with good quality decay data.

##### Acetylation site Occupancy Calculation

A SILAC experiment was conducted with HeLa cells grown in the heavy (H) condition treated with DMSO, and the light (L) condition treated with 5 mm aspirin-d_3_ for 8 h (Fig. 5*B*). A third SILAC condition (medium or M) was included, which was treated with 5 mm aspirin-d_3_ for 15 min. This was included in case of issues associated with requantification of acetylated peptides in H/L comparisons, but eventually acetylated peptide ratios were not required for occupancy calculation, so the M condition was unnecessary. This SILAC mixture is referred to as “occupancy mixture” or “Mix O.” The cells from Mix O and the resultant MS data were processed in parallel with those used for acetylation half-life analysis (described above). Acetylation site identifications from all mixtures (Mixes A to E and Mix O) were used to define a set of unmodified counterpart peptides as described in Fig. 5*A*. All unmodified counterpart peptides identified and quantified from Mix O were used to calculate acetylation site occupancy using equation 7. See Fig. 5*C* and 5*D* for derivation.

(Eq. 7) $$Occupancy\;\%=\left[1-\left(P_{r}/U_{r}\right)\right]\times 100$$

Where P_r_ and U_r_ are the H/L ratios for the total protein and unmodified counterpart peptide of each acetylated peptide. For reference, occupancy calculations were also applied to a control set of peptides not designated as unmodified counterparts. These were used as a presumptive negative control set with which the known unmodified counterparts could be compared.

##### Other Statistical Analyses

Survival assays were made with *n* = 4 replicates and differences between conditions measured by Student's nonpaired two tailed *t* test assuming equal variance. For acetylated lysine half-life analysis, the 1480 sites were divided into 6 subsections based on half-life using 6 h bins: 1, 0–6 h; 2, 6–12 h; 3, 12–18 h; 4, 18–24 h; 5, 24–30 h; and 6, >30 h. Annotation of known acetylation, ubiquitination and SUMOylation sites, GO terms, Pfam families and KEGG pathways were annotated to each site in Perseus using the most up-to-date annotations files and comparison with published data (28, 29). Relative enrichment of these in each sub-section was calculated by Fishers exact test in Perseus. Only values with a Benjamini-Hochberg FDR of <1% in at least one sub-section were used in the heatmap. Hierarchical clustering was performed using the Euclidian method calculating distances by averages using Perseus-reported enrichment factor scores for each category in each subsection.

Comparisons among subsections for significant differences between numerical means of protein half-life (from reference (30)) used the Student's *t* test (comparing each sub-section with an equally sized random sample of the entire set). The two-tailed test was employed using either a two-sample equal variance method, or two sample unequal variance method depending on whether the variance ratio was less or greater than 1.5 respectively.

For occupancy calculations, comparisons between the values calculated for true unmodified counterpart peptides, and those not thought to be unmodified counterparts, the two-tailed unpaired Student's *t* test was applied using Welch's correction.

## RESULTS

### 

#### 

##### Aspirin Cytotoxicity in HeLa Cells is Largely Independent of Protein Acetylation

**Fig. 1.**
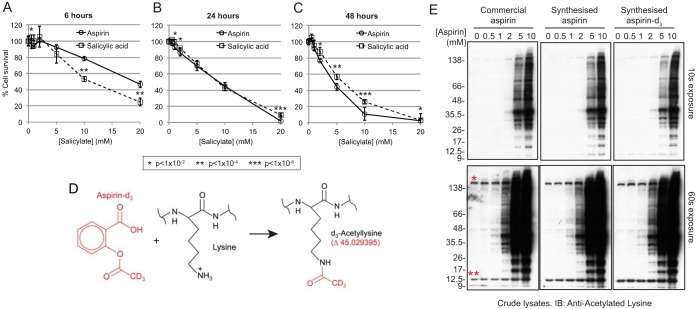
**Aspirin-d_3_ acetylates proteins in cultured cells.** *A–C*, Survival assays comparing aspirin and salicylic acid for death of HeLa cells over a range of concentrations from 0.5 to 20 mm, and over a range of durations; 6 h (A), 24 h (*B*), and 48 h (*C*). Four replicates were averaged and standard deviations are indicated as *error bars. t* test *p* values are indicated by *asterisks* (see below panel *B*). *D*, Schematic overview of the acetylation of the ε-amino-group on lysine side-chains by aspirin-d_3_. The acetyl-d_3_ group is distinguishable from the non-deuterated equivalent by ∼3 Da. *E*, Anti-acetylated lysine immunoblot analysis of crude cell lysates from HeLa cells exposed to the indicated concentrations of commercially sourced aspirin, in house synthesized unlabeled aspirin or in-house synthesized aspirin-d_3_ for 4 h. *Single* and *double asterisks* indicate positions of the most abundantly acetylated proteins in untreated cells (discussed further in relation to Fig. 3*A*).

The toxicity of aspirin to HeLa cells (human cervical cancer) was assessed by survival assay over a range of drug concentrations and exposure durations (Fig. 1*A*–1*C*). To try to specifically isolate the influence of acetylation on toxicity, comparisons were made with the non-acetylating metabolite of aspirin, salicylic acid (SA). After 6 h, greater than 90% cell survival was observed for aspirin concentrations of 5 mm or less (Fig. 1*A*). SA showed similar, if slightly higher toxicity over the same duration (Fig. 1*A*). Longer exposures to aspirin and SA caused increased cell death (Fig. 1*B*, 1*C*), although large differences between the two were not observed.

To establish the kinetics of aspirin uptake and hydrolysis under cultured cell conditions, aspirin and salicylic acid levels were monitored during 24 h exposure to 5 mm aspirin. The measurements fit well to a model whereby aspirin diffuses across the cell membrane more rapidly than salicylic acid (SA), and is hydrolyzed to SA around 200 times faster in the cell than the medium (supplemental Fig. S1*A*–S1*C*). As a result intracellular aspirin is consistently ∼40% of the medium concentration (supplemental Fig. S1*A*, S1*E*). Thus intracellular aspirin concentrations are closely linked to the extracellular concentration, and administration or removal of aspirin is relatively quickly mirrored by intracellular changes.

##### Unambiguous Definition of the Aspirin-mediated Lysine Acetylome in HeLa Cells

The addition of a single acetyl group (CH_3_CO) to a protein causes an increase in mass of 42.01 Da. To allow discrimination between endogenous acetylation and aspirin-mediated acetylation, labeled aspirin-d_3_ was synthesized in which the three hydrogen atoms of the acetyl group are substituted with deuterium (supplemental Fig. S2*A*–S2*C*). This does not affect aspirin reactivity, but resultant acetylations will be ∼3 Da heavier than endogenous equivalents (Fig. 1*D*), which is easily discriminated by modern mass-spectrometry instrumentation (supplemental Fig. S3).

Commercially obtained aspirin, in-house synthesized aspirin, and in-house synthesized aspirin-d_3_ displayed indistinguishable patterns of acetylated species in anti-AcK Western blots of cell lysates (Fig. 1*E*). It appears that dose has little influence on protein selection, as higher concentrations only increase the intensity of acetylated species, rather than change the pattern of conjugates (Fig. 1*E*).

**Fig. 2.**
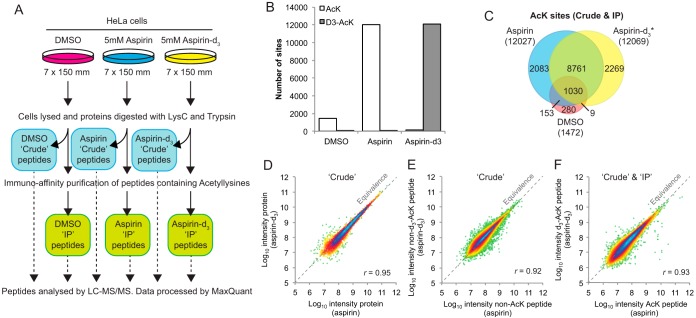
**Aspirin-d_3_ has identical lysine acetylation activity to isotopically typical aspirin but creates a unique acetylation signal.** *A*, Overview of the experiment to identify protein targets of aspirin-mediated lysine acetylation. *B*, Summary of numbers of AcK and d_3_-AcK sites identified in peptide preparations from the three cell treatments. *C*, Summary of overlap between experiments for identified acetylated lysine sites. * Only d_3_-Acetyllysines considered. Note; acetylation site lists from cells treated with unlabeled aspirin will contain both endogenous acetylated lysines as well as those acetylated by aspirin. *D*, Comparison between aspirin and aspirin-d_3_ treated cells for protein intensity in “Crude” extracts measured by mass spectrometry. *E*, As in *D*, except comparing the intensity of nonacetylated peptides. *F*, As in *D*, except comparing the intensity of AcK and d_3_-AcK peptides peptides in IPs from aspirin and d_3_-aspirin treated cells respectively. x = y line shown in *D*, *E*, and *F*.

To identify sites of acetylation a large-scale mass-spectrometry-based proteomics experiment was undertaken (Fig. 2*A*). Our study concentrated on acetylation of lysines because this PTM has well-established biological functions, and because the enrichment of nonlysine acetylated peptides is prohibited by the absence of suitable reagents. Three groups of cultured HeLa cells were exposed to 5 mm aspirin (unlabeled/isotopically typical form), 5 mm aspirin-d_3_, or vehicle control for four hours. This dose was chosen as it was non-toxic, gave a high level of acetylation (Figs. 1*E*, supplemental Fig. S4) and is representative of the concentrations used in previous cellular protein acetylation studies (12–14, 16, 19, 31–33). For each experimental condition a Lys-C/trypsin digest of unfractionated cell extract, designated 'crude' and an anti-AcK peptide preparation, made by immuno-precipitation (IP) of peptides using an anti-AcK antibody (17, 34, 35) were prepared. This allows both total proteome and acetylated peptide analysis (Fig. 2*A*) by LC-MS/MS (21, 22) searching for lysine modifications by acetylation (ΔMW 42.01) and d_3_-acetylation (ΔMW 45.03). Example spectra for both unlabeled and d_3_-labeled-acetylation of transaldolase at Lys^81^ are shown in supplemental Fig. S5.

After extensive filtering of the data (see Experimental Procedures for criteria), a total of 12069 aspirin-d_3_-mediated lysine d_3_-acetylation sites were identified in 3763 proteins (supplemental File. S1). Similar numbers of isotopically typical acetylation sites were found from cells treated with unlabeled aspirin (Fig. 2*B*). Only 94 d_3_-AcK sites were found in the unlabeled aspirin preps (Fig. 2*B*), where no d_3_-AcK sites should be expected, suggesting an actual false-positive rate in the order of 0.8%. There was a 73% overlap between labeled and unlabeled aspirin acetylation sites (Fig. 2*C*), consistent with the expected overlap between biological replicates of shotgun proteomics studies of this scale.

Untreated cells only yielded 1472 acetylation sites (Fig. 2*B*, 2*C*) with 70% overlap between this and the aspirin acetylome (Fig. 2*C*, 1039/1472 endogenous sites). Thus, aspirin targets a large proportion of lysine residues that are also endogenously modified. Unexpectedly, only 162 isotopically typical (endogenous) AcK sites were identified in preparations from aspirin-d_3_ treated cells (Fig. 2*B*), one-tenth of those found in DMSO treated cells. It is unlikely that aspirin administration suppressed endogenous acetylation, rather the MS analysis favored detection of the more abundant and numerous d_3_-acetylated peptides.

Semiquantitative comparisons between preps showed that both aspirin types gave broadly equivalent ion intensity data, confirming the labeled aspirin behaved identically to its unlabeled counterpart (Fig. 2*D*–2*F*).

##### Except for Histone N-terminal Tails, SMC3 and Enzymes Involved in Acetylation Aspirin Considerably Increases Existing Endogenous Acetylations

MS signal intensity correlates only weakly with absolute peptide abundance when comparing different peptides, but across a large mixed population, frequency distributions of peptide intensities are indicative of the dynamic range of abundance. Analysis of these data suggests that endogenously acetylated peptides are generally less abundant than aspirin-mediated acetylated peptides, but there is a small number of extremely abundant endogenous acetylations with peptide intensities three to four orders of magnitude higher than the median, that are not found acetylated to the same degree by aspirin (supplemental Fig. S6*A*–S6*C*). This is consistent with anti-AcK Western blots that show only a few apparently acetylated species in untreated cells, but a broad range in aspirin treated cells (Fig. 1*E*).

**Fig. 3.**
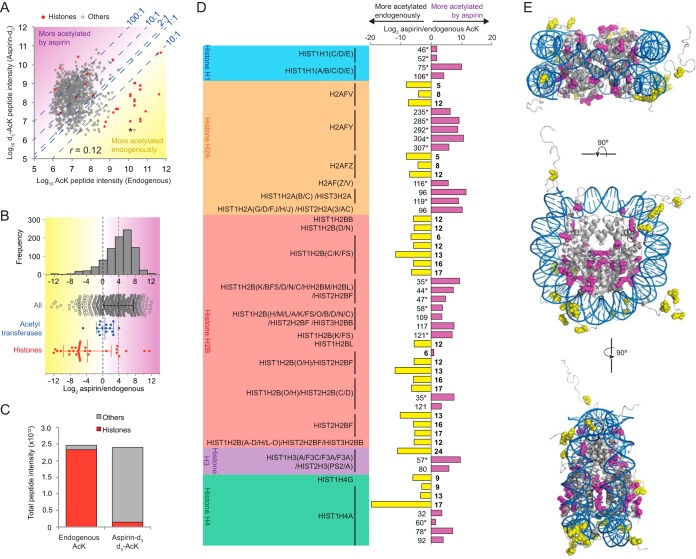
**Aspirin enhances acetylation site occupancy for the majority of endogenous sites excepting Histone N-terminal tails.** *A*, Scatter plot comparing intensities of acetylated peptides in DMSO treated cells (endogenous acetylation) with d_3_-acetylated peptides from aspirin-d_3_ treated cells. Total number of common sites was 1039, with 36 histone sites (*red*). *Asterisk* marks the acetylated peptide of K106 from SMC3. Note, scale is log_10_. Lines of equivalence (1:1) and intensity ratios of 2:1, 10:1, and 100:1 (aspirin-d_3_-AcK:endogenous AcK) are indicated. *B*, Ratio of acetylated peptide intensities for sites found both endogenously acetylated and acetylated by aspirin. *Upper portion* shows a frequency histogram of all ratios and lower section shows a Beeswarm plot of the same data including subsections for proteins involved in cellular acetylation and histone proteins. *C*, Proportion of total AcK peptide intensity derived from histone acetylation in untreated cells and d_3_-AcK peptide intensity from those treated with aspirin-d_3_. Note, non-log scale. *D*, Log_2_ values for the ratio aspirin-d_3_:endogenous AcK intensity for histone proteins. Ratios for peptides detected only in the aspirin-d_3_ treated cells were created by defining absent endogenous peptides with an intensity of 500,000, and are indicated by *asterisk* (*) *Magenta bars* are more acetylated with aspirin and *yellow* more acetylated endogenously. Residue numbers are indicaded, with bold representing those found in N-terminal tails. *E*, Mapping onto the structure of the nucleosome (PDB 1KX5) (53) of histone acetylation sites that are either more acetylated endogenously than by aspirin (*yellow*), or more acetylated by aspirin than endogenously (*magenta*). Modified lysines are shown with atoms as spheres with the remainder of the protein structure shown in gray schemtic format. DNA is shown in *blue*.

Direct comparisons between DMSO and aspirin-d_3_ treated preps for common (*n* = 1039) acetylated peptide signal intensity, allow evaluation of the relative abundance of each in the different conditions (Fig. 3*A*, 3*B*). This specifically addresses the question; to what extent does aspirin modify lysine residues that are also acceptors for endogenous acetylation? Peptides for which d_3_-AcK intensity is approximately the same as endogenous AcK intensity, will be found close to the 1:1 (equivalence) line in scatter plots (Fig. 3*A*) and with log_2_ ratios close to 0 (Fig. 3*B*). Those for which aspirin adds more than was already present will be found above the 1:1 line (Fig. 3*A*) or with positive log_2_ ratio values (Fig. 3*B* magenta), and those for which aspirin adds less than was already present, below the line (Fig. 3*A*) and with negative log_2_ ratio values (Fig, 3*B* yellow). These show that the vast majority of endogenously acetylated peptides were considerably more acetylated during aspirin treatment (on average 15 fold increased). Over 82% of endogenous acetylations, had d_3_-acetylated equivalents that were more than twice as abundant, 63% were 10-fold more abundant, and 22% of endogenous sites were more than 100-fold more acetylated in the presence of aspirin (Fig. 3*A*). As overall protein levels do not significantly change among preps (Fig. 2*D*), these results are explained by aspirin causing increased stoichiometry of acetylation at sites already acetylated in untreated cells.

In contrast to the general multifold amplification of endogenous acetylation by aspirin, some proteins have their acetylation signals less than doubled. In other words, aspirin adds fewer acetyl groups than was already present (Fig. 3*A* yellow). Among the rare group of proteins with ∼1:1 aspirin/endogenous acetylation are those which are themselves involved in acetyl group metabolism, such as acetylCoA synthetase, fatty acid synthetase, and N-acetyl transferase (Fig. 3*B*, blue). But the most striking set of proteins are those for which aspirin adds less than 10% of existing acetylation (Fig. 3*A* below 10:1 line), which contain a preponderance of histone sites (Fig. 3*A*, 3*B* red). In fact, of this group only one belongs to a non-histone protein (Fig. 3*A* asterisk), Lys^106^ from SMC3, which is known to be required for sister chromatid cohesion during S-phase of the cell cycle (36).

These observations are consistent with the idea that aspirin does not add a great deal more to the existing pool of acetylated protein if that protein is already highly acetylated under normal conditions. Indeed, by calculating the proportion of the total acetylated peptide signal intensity that is derived from histones (Fig. 3*C*), it is clear that histone acetylation makes up almost 90% of endogenous signal. In contrast, histone acetylations represent about 10% of the acetylated peptide signal intensity in aspirin treated cells. This suggests the two most abundant anti-AcK-reactive species found in extracts from untreated cells (Fig. 1*E* asterisks) are SMC3 (142kDa) and histones (11–22kDa) acetylations.

Although most histone acetylations are largely unaffected by aspirin, a few appear to have their acetylation site occupancy increased 10-fold or more by aspirin (Fig. 3*A*, 3*B*). Some are over 100 times more acetylated by aspirin, and 20 d_3_-acetylation sites that were detected in aspirin-d_3_ preps were not detected in preps from DMSO treated cells, suggesting trace or absent acetylation under normal conditions. Strikingly, it is the N-terminal tail regions that are least affected by aspirin, while all others are amplified considerably by aspirin (Fig. 3*D*, 3*E*). These sites are predominantly either buried in the nucleosome core structure or in regions contacting DNA.

##### Detection of an Aspirin-mediated Lysine Acetylation Depends Largely on the Cellular Abundance of the Protein

**Fig. 4.**
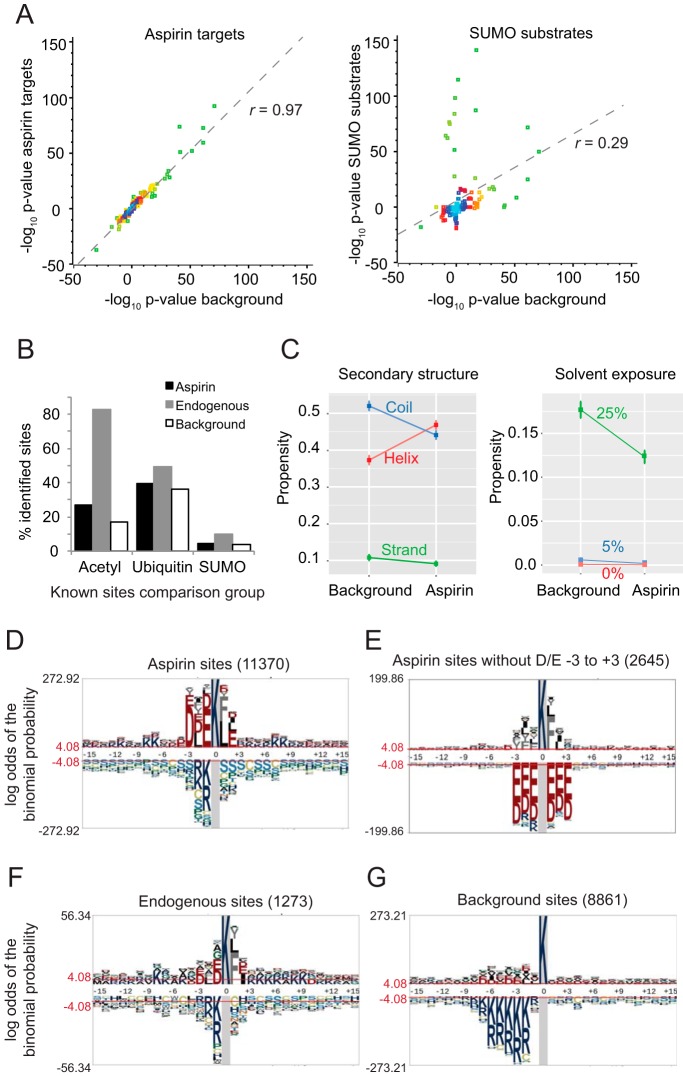
**The abundance of aspirin-mediated acetylations are linked to total protein abundance.** *A*, Comparison between a crude cell MS-based proteome (*background*) and proteins identified as aspirin acetylation targets for enrichment of GO terms (*left chart*). The same comparison for SUMO sites (*right chart*) is also made for reference purposes. GO analysis for cellular component, biological process, and molecular function was calculated using Panther (54), and each GO term is represented as a data point. Over-representations are plotted as positive values and under-representations as negative. Data best-fit lines (*broken gray*) and Pearson correlation coefficients are indicated. Data points are colored by density from *cyan* (high density), through *blue*, *red* and *yellow*, to *green* (low density). *B*, Comparisons of the aspirin lysine acetylome with other large scale lysine PTM studies; endogenous acetylation, endogenous ubiquitination and exogenous SUMOylation (data from phosphosite plus (37) and references (28, 29). *C*, Comparison of Jpred (38) predicted secondary structure propensity (*left*), and predicted solvent exposure (*right*) between the aspirin lysine acetylome and a background control group of lysine-containing peptides detected from a crude HeLa extract proteome. The majority of lysines in both groups are predicted to be >25% solvent exposed. *D–G*, pLogo site analysis (24) for the indicated groups of lysines found acetylated by aspirin (*D*, *E*), endogenously modified (*F*) or the background set of lysines described in *C* (*G*). *p* value <0.05 cutoff is shown broken red. Note different *y* axis scales.

The 12,069 aspirin-AcK sites identified in this study were identified from 3763 proteins, and 1472 endogenous AcK sites were identified in 746 proteins. Comparing site frequency per protein (supplemental Fig. S7*A*, S7*B*) shows that less than half of the aspirin targeted proteins had only a single detectable site, whereas two-thirds of the endogenous group had only a single site identified. Also, almost 5% of proteins were found to have more than ten aspirin acetylation sites, whereas only 0.6% of proteins from untreated preps were similarly modified. This group of aspirin hyper-acetylated proteins are commonly involved in cell structure; Plectin (105 sites), myosin-9 (64 sites), Filamin-A and B (56 and 44 sites), AHNAK/desmoyokin (52 sites), and dynein 1 heavy chain 1 (45 sites), which are among the most abundant proteins in the cell. Indeed, estimates of copy-number per cell (23), correlate with acetylated peptide intensity better for the aspirin data than the endogenous data (supplemental Fig. S7*C*, S7*D*), indicating that the more abundant a protein is in the cell, the more likely that its acetylated peptides are detected in shotgun proteomics studies such as this. Consistent with this, proteins detectably acetylated by aspirin do not show any significant enrichment for functional group over a control list of proteins derived from shotgun MS analysis of the crude HeLa cell lysate (Fig. 4*A*, *left*). This is in contrast with a SUMOylation site list prepared from the same cell type, which shows some considerable differences between crude and purified data sets (Fig. 4*A*, *right*).

Many PTMs have been studied on the proteomic scale giving over 30,000 acetylation, 60,000 ubiquitination and 5000 SUMOylation sites (PhosphoSitePlus^®^, www.phosphosite.org, (28, 29, 37)). Comparisons with these (Fig. 4*B*, supplemental Fig. S8), show that the aspirin acetylation sites overlap more with ubiquitination sites than known acetylation sites. This contrasts with the “endogenous” acetylation sites detected in this study, which as expected shows greater overlap with known acetylation sites than ubiquitination sites. The precise statistical significances of these intersections cannot be calculated without knowledge of the total detectable proteome, but the data so far imply that aspirin appears to have not only the potential to amplify endogenous acetylation signals, but also to interfere with protein stability.

Secondary structure and solvent exposure comparisons (38) show a significant overrepresentation of helical regions (Fig. 4*C* left), and an underrepresentation of buried lysines (Fig. 4*C* right) in the aspirin acetylome compared with a background lysine set. Solvent exposure will be critical for acetylation by a chemical, but the reason for the apparent bias toward helical domains is presently unclear. Sequence analysis (24) shows aspirin acetylations are commonly found within acidic domains, with aspartic acid and glutamic acid residues at positions −3 and −1 being most significantly overrepresented (Fig. 4*D*–4*G*). Aspirin acetylation sites lacking acidic residues within three amino-acids of the target lysine (2645 sites uploaded) generate a logo with an over-representation of leucine, phenylalanine and tyrosine in the +1 position, as is common to endogenous sites (1273 sites uploaded) (Fig. 4*E*, 4*F*). It may be that a sub-population of aspirin-mediated acetylations share a functional link with endogenous acetylations, perhaps by recycling of aspirin acetyl groups into endogenous acetylation systems. Further sequence analysis shows some interdependence between amino-acids proximal to acetylated lysines, most strikingly the relationship between leucine and phenylalanine in the +1 position and acidic residues in the −1 and −3 positions (supplemental Fig. S9). Acetyl phosphate (AcP) chemically acetylates lysines in bacteria (39), and sequence analysis of published sites also shows a similar propensity for acidic regions (supplemental Fig. S10). Together these data confirm aspirin acetylates in a non-enzymatic fashion expressing no particular preference for targets based on cellular location or function.

##### Aspirin Acetylation Site Occupancies Are Low

**Fig. 5.**
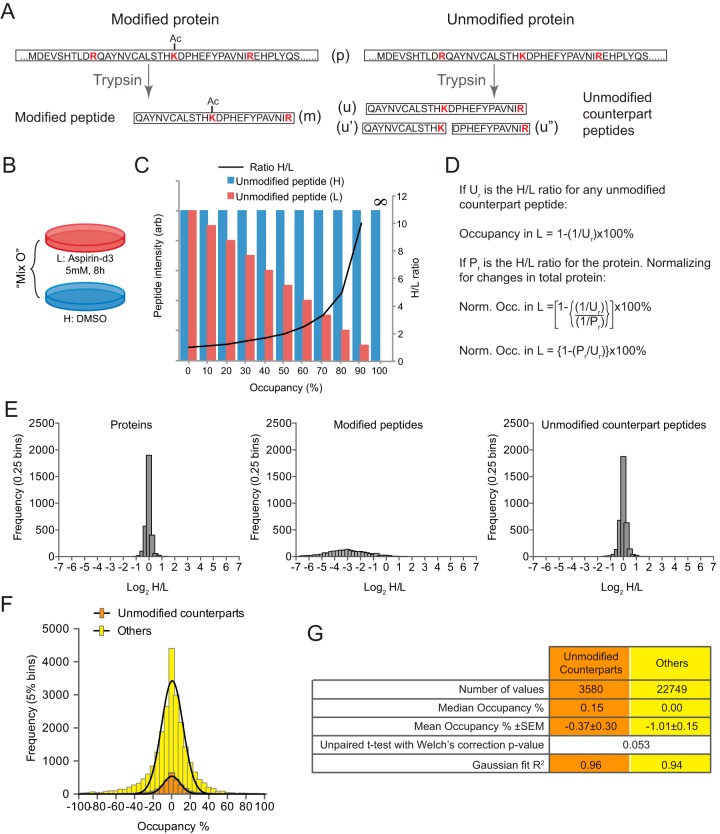
**The lysine site occupancy of aspirin-mediated acetylations is very low.** *A*, For a protein (p) acetylated at lysine (K), digestion by trypsin yields a modified peptide (m). The unmodified protein can yield two unmodified counterpart peptides (u′ and u″), and if cleavage after the target lysine is missed, another unmodified counterpart peptide (u). *B*, A SILAC experiment designed to allow aspirin-mediated lysine acetylation site occupancy calculation. *C*, Example of relative abundances of heavy and light forms of an unmodified counterpart peptide for modifications at different % occupancy expected from the experiment shown in *B. Solid black line* shows the resultant H/L ratios at each % occupancy example. *D*, An equation to determine aspirin-mediated acetylation occupancy from the experiment shown in *B. E*, Frequency distributions of log_2_H/L ratios for total protein, unmodified counterpart peptides and modified peptides from the experiment shown in part *B*. Modified peptide ratios are generated by MaxQuant requantification due to zero intensity in the DMSO treated condition. Note, high occupancy sites would yield positive values for u, u′ and u″ peptide log_2_H/L ratios. *F*, Frequency distributions of occupancy calculations based on the data shown in part *E* (*orange*) in comparison with a control set of peptides thought not to be unmodified counterparts (*yellow*). *G*, Statistical analysis from data presented in *F*. Although median and average occupancies are calculated to be fractionally higher for counterpart peptides than non-counterparts, the difference is not statistically significant, indicating that for the vast majority of acetylated lysines, occupancy is so low as to be immeasurable by this analysis.

Although we can identify sites of acetylation, and compare relative abundance between aspirin acetylations and endogenous equivalents, it is important to know the proportion of a protein that is acetylated by aspirin. This is critical for the evaluation of the potential to alter protein function either by introduction of a new chemical signal or by competition with other PTMs. Attempts to determine site occupancy or stoichiometry for phosphorylations have shown them to be very high, with median values of between 50 and 90% for a variety of kinase substrates (40), while protein acetylation in yeast and human cells is reported to be low, in the 0–10% range (41, 42). Proteomic-based occupancy calculations rely heavily on changes in unmodified counterpart peptide (Fig. 5*A*) abundance during a stimulation or treatment that alters modification status. As such, calculations of site occupancy are more accurate when the difference in modification between the two experimental conditions is large. As there is no aspirin-mediated acetylation in absence of aspirin, a SILAC experiment was designed to compare untreated cells with those treated with aspirin (Fig. 5*B*) that allows occupancy calculation using only unmodified peptide and total protein ratios (Fig. 5*C*, 5*D*). However, even with high quality SILAC data, changes in unmodified counterpart peptides were so small (Fig. 5*E*) that calculations often failed to provide a positive value for occupancy (supplemental File S2). Frequency distributions of aspirin acetylation occupancy values were normally distributed around 0.15% (Fig. 5*F*). Indeed, the calculated aspirin-mediated acetylation occupancies were not significantly different from those of a control set of peptides not identified as unmodified counterpart peptides (Fig. 5*F*, 5*G*). This implies that aspirin acetylation affects only a very small proportion of the total pool of any protein, with median occupancy being less than 1%.

##### Aspirin-mediated Lysine Acetylations Have a Wide Range of Half-lives in Cultured Cells

**Fig. 6.**
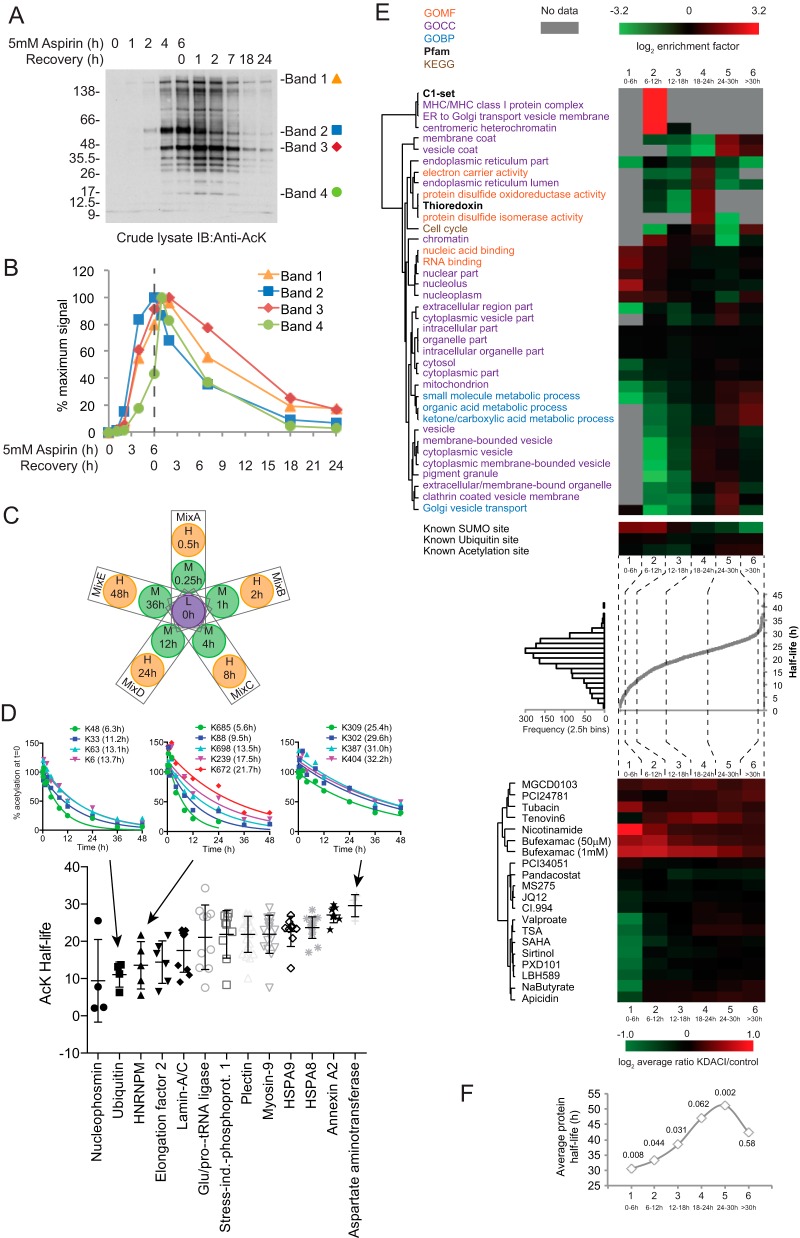
**Half-lives of aspirin-mediated lysine acetylation signals.** *A*, Anti-AcK immunoblot of 6.25 μg (protein) crude cell lystes from HeLa cells treated with 5 mm aspirin for the indicated times, before change to medium lacking aspirin (recovery). Bands selected for semi-quantitative analysis in *B*, are indicated. *B,* Densitometry quantitation of the data shown in part *A* for four selected bands. *C*, Design of a SILAC experiment to study half-lives of lysine acetylations by aspirin. L- light, M- medium and H - heavy lysine/arginine isotope containing culture medium. *D*, Half-lives of selected proteins with multiple site data. *E*, Analysis of 1480 aspirin acetylation sites data by separation into six sub-sections (1, 0–6 h, *n* = 47; 2, 6–12 h, *n* = 135; 3,12–18 h, *n* = 287; 4, 18–24 h, *n* = 541; 5, 24–30 h, *n* = 421; and 6, >30 h, *n* = 49) on the basis of half-life as shown in the central scatter plot and lateral histogram. Large upper heatmap shows hierarchial clustering of GO, Pfam and KEGG terms enriched in the six sub-sections. Only groups with a Benjamini-Hochberg FDR <1% in at least one subsection were included in the figure. Multiple sites from the same protein were considered as separate entries. Enrichments calculated in Perseus using Fishers exact test. Small, upper heatmap shows the same analysis for enrichment of sites already described as being modified by SUMO-2, ubiquitin or acetylation. Lower heatmap shows hierarchical clustering of sub-section average log_2_ lysine deacetylase inhibitor KDACI/control ratios for lysine sites identified in common between this study and those endogenous acetylations described in ref (43). *F*, Scatter plots show average protein half-life per subsection as described in reference (30). *p* values according to *t* test comparisons with equally sized control groups of randomly selected data are indicated.

Although aspirin-mediated acetylations are know to persist for hours after removal of the drug (18), the dynamics on the individual site level have not been explored. Anti-AcK western-blot analysis suggests that even 24 h after exposure, some aspirin acetylations are still present (Fig. 6*A*, 6*B*). A time-resolved SILAC experiment was undertaken to quantitatively monitor AcK peptide abundance after drug withdrawal, and derive an acetylation half-life for each site. In the experiment a “Light” labeled SILAC reference sample was prepared from cells treated with 5 mm aspirin-d_3_ for 8 h. Ten further cultures of “Medium” and “Heavy” labeled cells were also incubated with 5 mm aspirin-d_3_ for 8 h before the culture medium was replaced with medium lacking aspirin, for varying lengths of time prior to harvesting (Fig. 6*C*). Thus, it was theoretically possible to obtain quantitative information on acetylation sites with 10 data points over a 48 h time-course. In practice, only 1480 sites provided good enough data to fit to an exponential decay model and yield reliable half-life values (supplemental File S3). These data encompassed a large range of half-lives from 1 h 23 min for acetylation at Lys^49^ of mitochondrial ATPase inhibitor ATPIF1, to 40 h 24 m for acetylation at Lys^215^ of mitochondrial malate dehydrogenase MDH2. The median half-life was ∼21 h. Growth retardation is likely to explain why around a third of half-lives were calculated as greater than the typical doubling time of HeLa cells of 24 h.

Examples of raw data for three multiply modified proteins and overall data for thirteen proteins are shown in Fig. 6*D*. Nucleophosmin, which is thought to have roles in nucleosome structure, ribosome biogenesis, genomic stability, apoptosis, p53 signaling, and centrosome duplication, contains two of the three shortest half-life sites at Lys^141^ and Lys^266^ (2 h 4 m and 2 h 15 m). It also has two sites with longer half-lives at Lys^257^ and Lys^32^ (7 h 46 m and 25 h 32 m). In contrast, other multiply-acetylated proteins such as HSPA8, HSPA9, Annexin A2, and Asparate aminotransferase displayed acetylation half-lives within a 7h range (Fig. 6*D*). To determine whether there is any broader relationship between aspirin-mediated lysine acetylation half-life and protein function a GO, KEGG and PFam enrichment analysis was undertaken. Despite the large number of categories considered, only a small number showed significant enrichment in any single subsection. These included acetylation of MHC complex components β-2-microglobulin (Lys^61^ and Lys^68^) and HLA class I histocompatibility antigen (Lys^145^ and Lys^151^) with half-lives of less than 12 h. Mitochondrial proteins tended to be over-represented in long half-life groups, as were, to a lesser degree vesicular proteins (Fig. 6*E* upper heatmap). Proteins from the nucleus and nucleolus and those associated with chromatin tended to have low acetylation persistence (Fig. 6*E* upper heatmap), implying relatively rapid reversal in these compartments.

Two active biological processes will impact on the rate of decay of an acetylation signal in cultured cells; the rate of protein turnover, and the action of cellular lysine deacetylases (KDACs). Two proteomic studies measuring protein turnover rate (30) and assessing the influence of KDAC inhibitors on endogenous acetylation (43) were compared with the acetylation half-life data (Fig. 6*E* lower heatmap and Fig. 6*F*). This showed that short half-life sites were commonly sensitive to the HDAC6 inhibitor bufexamac (44), and the sirtuin inhibitor nicotinamide (45). Protein turnover rate has a significant, but weaker influence upon acetylation half-life (Fig 6*F*), although for the majority protein turnover half-life is much longer than acetylation half-life (supplemental Fig. S11). SUMO sites that are also acetylated by aspirin tend to lose acetylation quickly (Fig. 6*E* small upper heatmap), which is possibly a consequence of the fact SUMO substrates are typically nuclear, a cellular location rich in proteins with short acetylation half-lives (Fig. 6*E*). Conversely, known acetylation sites are more common in the long half-life sub-group, probably because endogenous acetylations will have the same half-lives as aspirin-mediated ones, and slow removal makes identification in proteomics experiments more likely.

##### Bufexamac Enhances Aspirin-mediated Cytotoxicity

**Fig. 7.**
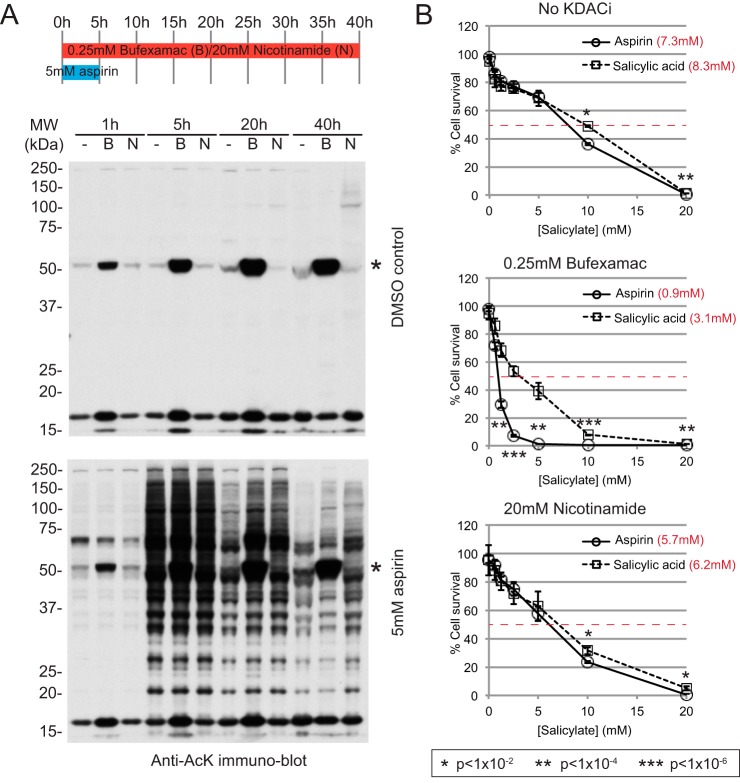
**Bufexamac enhances aspirin-mediated cytotoxicity.** *A*, Upper schematic shows experimental timings. In six-well-plates, ∼40% confluent HeLa cells were exposed to either 5 mm aspirin, or DMSO control, along with the KDAC inhibitors bufexamac (0.25 mm) or Nicotinamide (20 mm) or DMSO control (-). Aspirin treatment was ceased after 5 h by replacment of media for that containing only the KDACi drugs. For each well, cells were lysed in 210 μl Laemmli's sample buffer plus 0.7 m 2-mercaptoethanol, before boiling and sonication. 30 μl of each sample was fractionated on a denaturing 10% polyacrylamide gel before immunoblotting for acetylated lysines as described under M&M. Equal volume loading rather than equal protein loading was used to avoid the complication of acetylated lysine signal dilution by differential cell growth rates caused by KDAC inhibitors. Asterisk (*) species is most likely to be acetylated tubulin. *B*, HeLa cell survival assays comparing aspirin and salicylic acid for cytotoxicity over a range of concentrations from 0.63 to 20 mm, over 24 h incubation. Cells were exposed to no KDAC inhibitor, or 0.25 mm bufexamac, or 20 mm nicotinamide. Four replicates were averaged and standard deviations are indicated as *error bars. t* test *p* values comparing aspirin with salicylic acid are indicated by *asterisks*. Calculated concentrations required to kill 50% of cells are indicated in *red*.

To explore the influence of bufexamac and nicotinamide on aspirin-mediated protein acetylation, protein acetylation and cytotoxicity in HeLa cells was investigated. Both bufexamac and nicotinamide delay deacetylation in cells exposed to 5 mm aspirin for 5 h (Fig. 7*A*). Bufexamac (0.25 mm) significantly increases aspirin-mediated cytotoxicity, lowering the concentration of aspirin required to kill 50% of cultured cells from 7.3 mm to 0.9 mm (Fig. 7*B* middle chart). Under the same conditions, it only modestly reduces salicylic acid cytotoxicity from 8.3 mm to 3.1 mm. Nicotinamide has a small and equal effect on the toxicity of both aspirin and salicylic acid (Fig. 7*B*, lower chart). This result is consistent with KDAC inhibition sensitizing cells to the toxic affects of aspirin-mediated protein acetylation. These results are consistent with previous work showing synergy between aspirin and the KDAC inhibitors suberoylanilide hydroxamic acid and sodium butyrate in induction of ovarian cancer cell death (46).

## DISCUSSION

The discovery that aspirin functions as an NSAID by acetylation of COX enzymes (7, 8), created a paradigm for the action of aspirin: Acetylation of a specific protein leads to a biologically relevant outcome. This model has been expanded to explain other functions of the drug via acetylation of intra- and extra-cellular proteins (8–10, 12–14, 16, 19, 31–33, 47). Proteomics approaches using cultured cells and modified forms of the drug have shown that aspirin's ability to acetylate extends beyond a handful of proteins (14, 18, 19), but critical details such as the relative scale of aspirin-mediated acetylation and site stoichiometry were not explored.

We have used isotopically labeled aspirin-d_3_, in combination with peptide level anti-AcK antibody affinity purification and current LC-MS/MS instrumentation to identify over 12000 sites of aspirin-mediated lysine acetylation from cultured human cells. The majority of endogenous lysine acetylations are greatly amplified by aspirin, often by orders of magnitude, but despite this, site stoichiometry is low by comparison with other signaling PTMs such as phosphorylation (40). Even after extended exposures to millimolar quantities of aspirin, as is routinely used in functional studies, standard SILAC proteomic methods are unable to accurately calculate stoichiometry because of immeasurably small changes in unmodified counterpart peptide abundances. In fact our data imply that lysine acetylation stoichiometry, even during exposure to high doses of aspirin, are for the majority, less than 1%. This apparent paradox between large signal amplification and low occupancy is explained by the fact that endogenous acetylations are for most, already at very low stoichiometry, being typically much lower than 1% (41, 42). Thus, aspirin is simply amplifying what is already a very low signal.

Conversely, aspirin did not greatly amplify the acetylation of proteins that are already known to be important targets for endogenous acetylation. Specifically, the modification of enzymes involved in the cellular acetylation system, the N-terminal tails of histones and SMC3 were all only modestly affected by aspirin. It has been noted that endogenous acetylation stoichiometry correlates closely with apparent biological significance (41). It is therefore the case that those proteins with relatively high endogenous acetylation are not substantially altered after exposure of cells to aspirin. Indeed, proteomic studies in yeast and bacteria reveal that low stoichiometry endogenous acetylations are likely to be nonenzymatic “lesions” caused by interactions between proteins and reactive metabolites such as acetyl-CoA (41) and AcP (39) (and reviewed in (48)). These observations are entirely consistent with this study using aspirin, which is also acting as an apparently unspecific acetylating chemical. It is thus likely that a major role of lysine deacetylases is to remove this “chemical noise” from the proteome. This supports the model of a two-tiered cellular acetylation landscape, with one group of relatively high stoichiometry and biological relevance, and the other of low stoichiometry and relevance. The former are more likely to be specifically generated by acetylating enzymes, while the latter may be predominantly of chemical origin.

These findings suggest that without knowledge of acetylation site stoichiometry, the biological importance of previously identified aspirin-mediated protein acetylations (12–14, 19, 31–33) is difficult to assess. Certainly, low stoichiometry almost completely excludes the possibility that aspirin can compete with other lysine-specific PTMs such as ubiquitin. However, it cannot be excluded that low stoichiometry may still lead to an important biological outcome, it is likely this would only exist in exceptional circumstances as cells tolerate aspirin-induced, large-scale low occupancy acetylations very well. It is also possible that in rare cases, aspirin could give rise to high occupancy or high persistence acetylation. Taking the COX enzymes as examples, it appears that an inherent affinity for the aspirin molecule itself contributes toward the direction of the chemical toward the active site (49). Furthermore, aspirin-mediated serine acetylation, as in the case for COX enzymes, may be resistant to deacetylation because endogenous enzymes are incapable of reversing the modification. Notably aspirin-mediated serine acetylations seem to be rare (19), suggesting they are not as reactive as lysines to acetylating chemicals. Aspirin acetylations are commonly found within regions containing acidic amino acids. A lysine within a localized negative charge environment may have a lower ε-amino-group p*K_a_*, tending more toward the deprotonated form under physiological conditions. Deprotonation is required for nucleophilic attack by acetylating chemicals (50).

For this study we used relatively high doses of aspirin (4–5 mm), although such exposures are not uncommon for acetylation studies on cultured cells (12–14, 16, 19, 31–33). We expect that different doses are more likely to affect site occupancy than alter site selectivity. Indeed, anti-AcK Western-blots show different doses alter signal intensity rather than species modified. Due to its hydrolysis in biological solutions, different tissues will be exposed to different concentrations of aspirin after administration. Although circulating plasma levels of aspirin have been measured to be to be around 50–300 μm after a typical analgesic dose of 300–600 mg (51, 52), gut epithelia may be exposed to millimolar concentrations, while low perfusion tissues will encounter less. It is therefore reasonable to assume that in the context of a whole organism, cellular protein acetylation by aspirin will depend largely on the balance between the local concentration of the drug, and the activity of cellular deacetylases. Whole organism acetylation studies will be revealing in this context.

In conclusion, this work shows that although aspirin-mediated acetylation of proteins on lysine side-chains is spread widely across the expressed proteome, it is mostly at a low level. Scavenging lysine deacetylases work to limit aspirin's potential to interfere with canonical acetylation signaling pathways, and blunt its ability to influence biological processes.

## Supplementary Material

Supplemental Data

## Footnotes

^1^ The abbreviations used are:

ASA: Acetyl salicylic acid
NSAID: Nonsteroidal anti-inflammatory drug
COX: Cyclooxygenase
PTM: Post-translational modification
DMEM: Dulbecco's Modified Eagle's Medium
PBS: Phosphate buffered saline
LC-MS/MS: Liquid chromatography tandem mass-spectrometry
SILAC: Stable isotopic labeling of amino-acids in cell culture
SA: Salicylic acid
NMR: Nuclear magnetic resonance
HRP: Horse radish peroxidase
DMSO: Dimethylsulfoxide
Tris: Tris(hydroxymethyl)aminomethane
HCl: Hydrochloric acid
DTT: Dithiothreitol
LysC: Endoproteinase Lys-C
TFA: Trifluoroacetic acid
NaCl: Sodium Chloride
AcK: Acetylated lysine
LFQ: Label free quantification
GO: Gene ontology
KEGG: Kyoto Encyclopedia of Genes and Genomes
Pfam: Protein families database
KDAC: Lysine deacetylase.
