# Supplementary material for: A Proteomic Approach to Analyze the Aspirin-mediated Lysine Acetylome
Source: Mol Cell Proteomics. 2016 Dec 5;16(2):310–26. doi: 10.1074/mcp.O116.065219 (PMC5294217; doi:10.1074/mcp.O116.065219)
Supplement: Supplemental Data [file supp_16_2_310__index.html]

A proteomic approach to analyse the aspirin-mediated lysine acetylome — A Proteomic Approach to Analyze the Aspirin-mediated Lysine Acetylome — Aspirin-mediated Lysine Acetylation — Supplemental Data 

# A Proteomic Approach to Analyze the Aspirin-mediated Lysine Acetylome

## Supplemental Data

- Supplementary figures (.pdf, 8.5 MB) - Online supplementary figures
- Supp. file 1 (.xlsx, 36.4 MB)
- Supp. file 2 (.xlsx, 18.7 MB)
- Supp. file 3 (.xlsx, 21.5 MB)
