# Supplementary material for: A Proteomic Approach to Analyze the Aspirin-mediated Lysine Acetylome
Source: Mol Cell Proteomics. 2016 Dec 5;16(2):310–26. doi: 10.1074/mcp.O116.065219 (PMC5294217; doi:10.1074/mcp.O116.065219)
Supplement: Supplemental Data [file 10.1074_O116.065219_mcp.O116.065219-1.pdf]

**Supplementary information**

# A proteomic approach to analyse the aspirin-mediated lysine acetylome.

Michael H. Tatham[1], Christian Cole[2], Paul Scullion[3], Ross Wilkie[4,5], Nicholas J Westwood[4], Lesley A. Stark[6], Ronald T. Hay*[1]

## Supplementary datasets.

Supplementary file 1. Excel workbook containing the data pertaining to the experiment described in figure 2A (identification of aspirin-medicated acetylation sites from cultured human cells). The first worksheet ("sheet details") describes the contents of each worksheet in the workbook.

Supplementary file 2. Excel workbook containing the data pertaining to the experiment described in figure 5 (calculation of aspirin-mediated lysine acetylation site stoichiometry). The first worksheet ("Information") describes the contents of each worksheet in the workbook.

Supplementary file 3. Excel workbook containing the data pertaining to the experiment described in figure 6C (calculation of aspirin-mediated lysine acetylation site signal half-life). The first worksheet ("sheet details") describes the contents of each worksheet in the workbook.

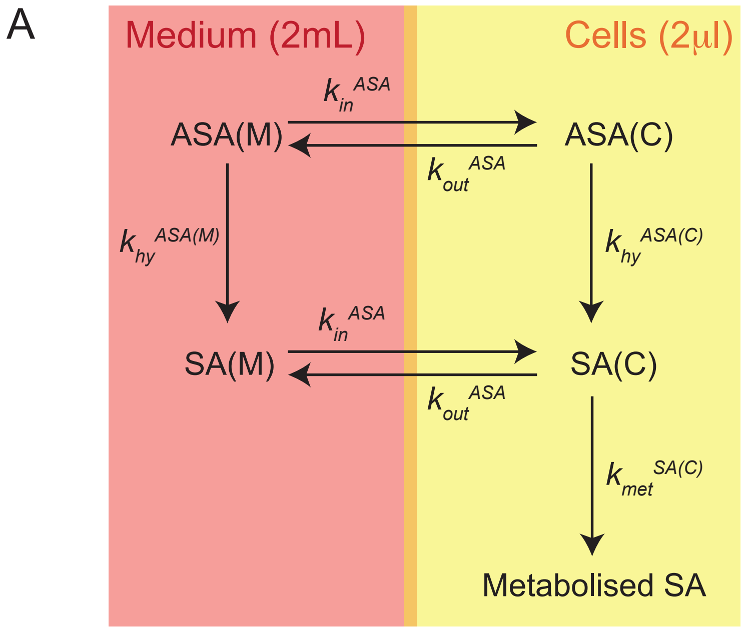


C

| Parameter | Value | Units |
|---|---|---|
| $k_{in}^{ASA}$ | 0.0132 | mL/h |
| $k_{out}^{ASA}$ | 0.0132 | mL/h |
| $k_{in}^{SA}$ | 0.0075 | mL/h |
| $k_{out}^{SA}$ | 0.0075 | mL/h |
| $k_{hy}^{ASA(C)}$ | 10.38 | L/h |
| $k_{hy}^{ASA(M)}$ | 0.0486 | L/h |
| $k_{met}^{SA(C)}$ | 7.881 | L/h |
| $[ASA(M)]_0$ | 5.439 | mM |
| $[SA(M)]_0$ | 0.3744 | mM |

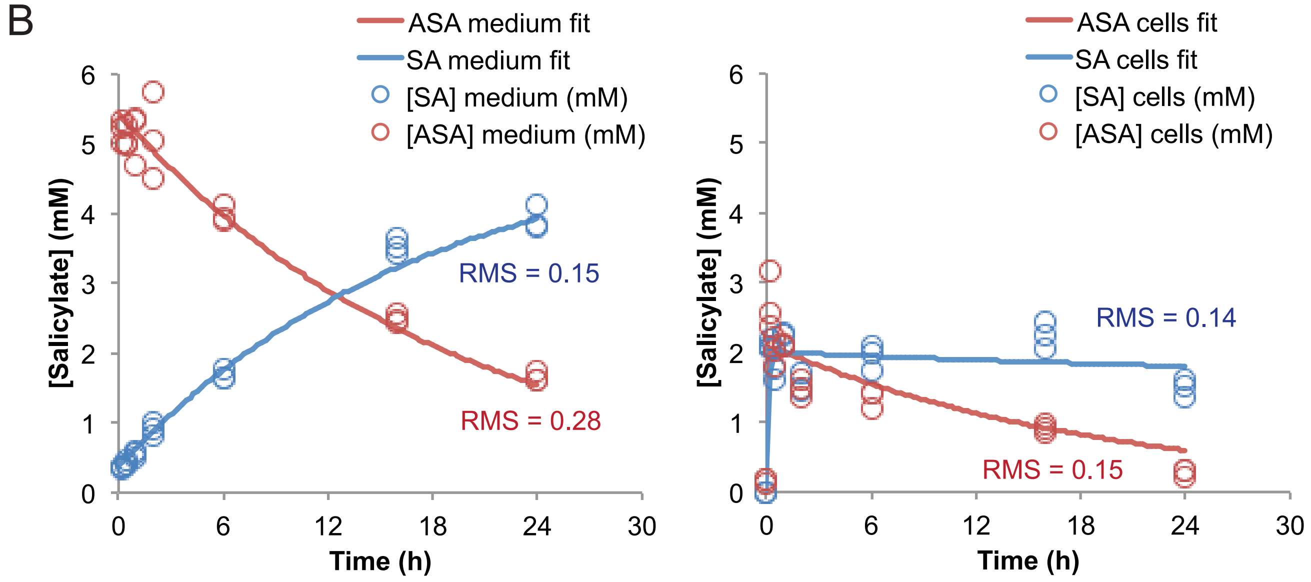


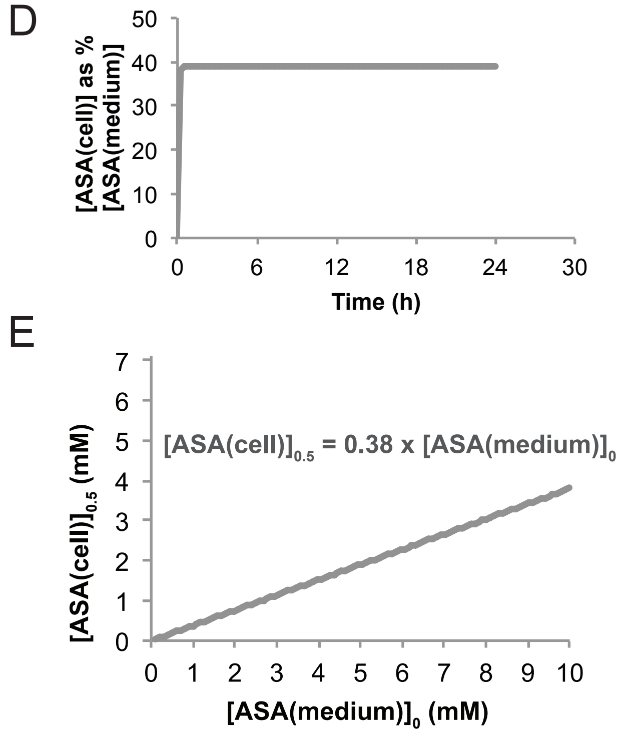


**Figure S1. Kinetic model for aspirin uptake and hydrolysis in cultured cells.** A. Reaction mechanisms defining the diffusion of aspirin (ASA) between culture medium and HeLa cells, and for the hydrolysis of ASA into salicylic acid (SA) in both 'compartments'. The system also requires a mechanism for the metabolism of cellular salicylic acid to fit to the experimental data, although the biological details are unclear. Note also that although the hydrolysis of ASA is defined by a single rate it is likely to involve multiple processes such as the activity of esterases and the acetylation of proteins, so the constant will be an apparent constant derived from multiple individual reactions. B. Fit of a model (solid lines) to experimental data (circles) for concentrations of ASA and SA in culture medium (left) and cells (right). RMS deviations of the model fit are indicated. C. Kinetic rate constants derived for the fit of the model to the experimental measurements of ASA and SA in culture medium and cells (B). D. Chart of [ASA(cell)] as a % of [ASA(medium)] during the modeled time-course E. Predicted relationship between the starting concentration of aspirin in the medium and the peak concentration in cells (at time t=0.5h). Equation of the relationship is shown. These data predict that aspirin concentration in cells is around 40% of the medium concentration over time and a range of starting concentrations in the medium.

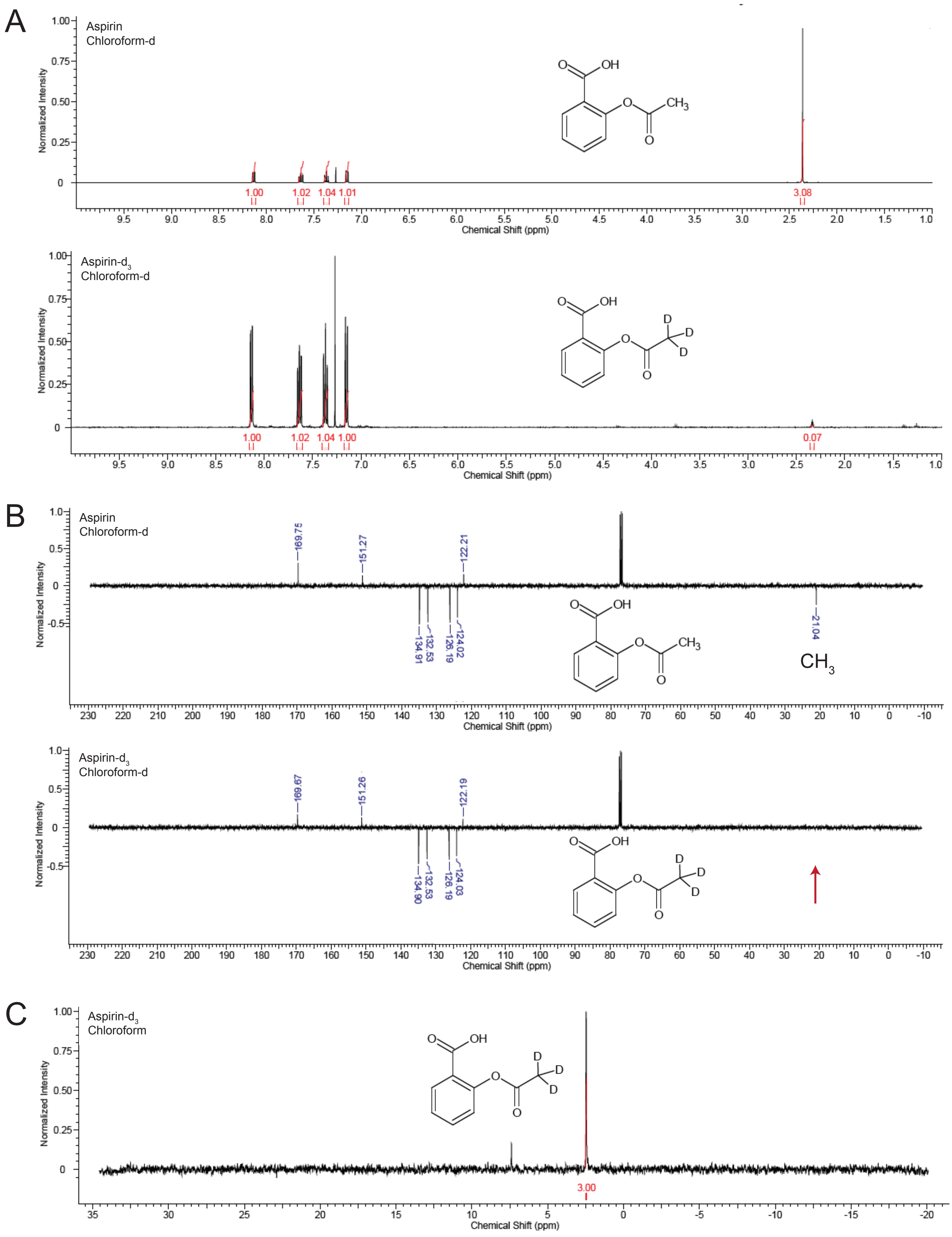


**Figure S2. Confirmation of aspirin-d$_3$ synthesis by NMR spectroscopy.** A. $^1$H NMR spectra of in-house synthesised aspirin (upper) and aspirin-d$_3$ (lower). B. $^{13}$C NMR spectra of in-house synthesised aspirin (upper) and aspirin-d$_3$ (lower). C. $^2$D spectrum of in-house synthesised aspirin-d$_3$.

In the $^{13}$C spectrum of aspirin, the ester carbon and the carboxylic acid carbon have very similar chemical shifts (~169.7 ppm) and so appear as one signal. Hence only 8 signals are visible in the spectrum and not the expected 9 signals.The $^1$H spectrum of aspirin-d$_3$ has the correct aromatic signals and negligible signal for the methyl group suggesting each hydrogen has been replaced by a deuterium. This is further confirmed by the deuterium NMR as it shows one signal ~2.5 ppm which is typical for a $CD_3$ functionality. In the $^{13}$C spectrum of aspirin-d$_3$ the signal for the $CD_3$ is absent, however this is to be expected. The signal for the $CD_3$ will be there, it is just particularly weak and will be lost in the noise.

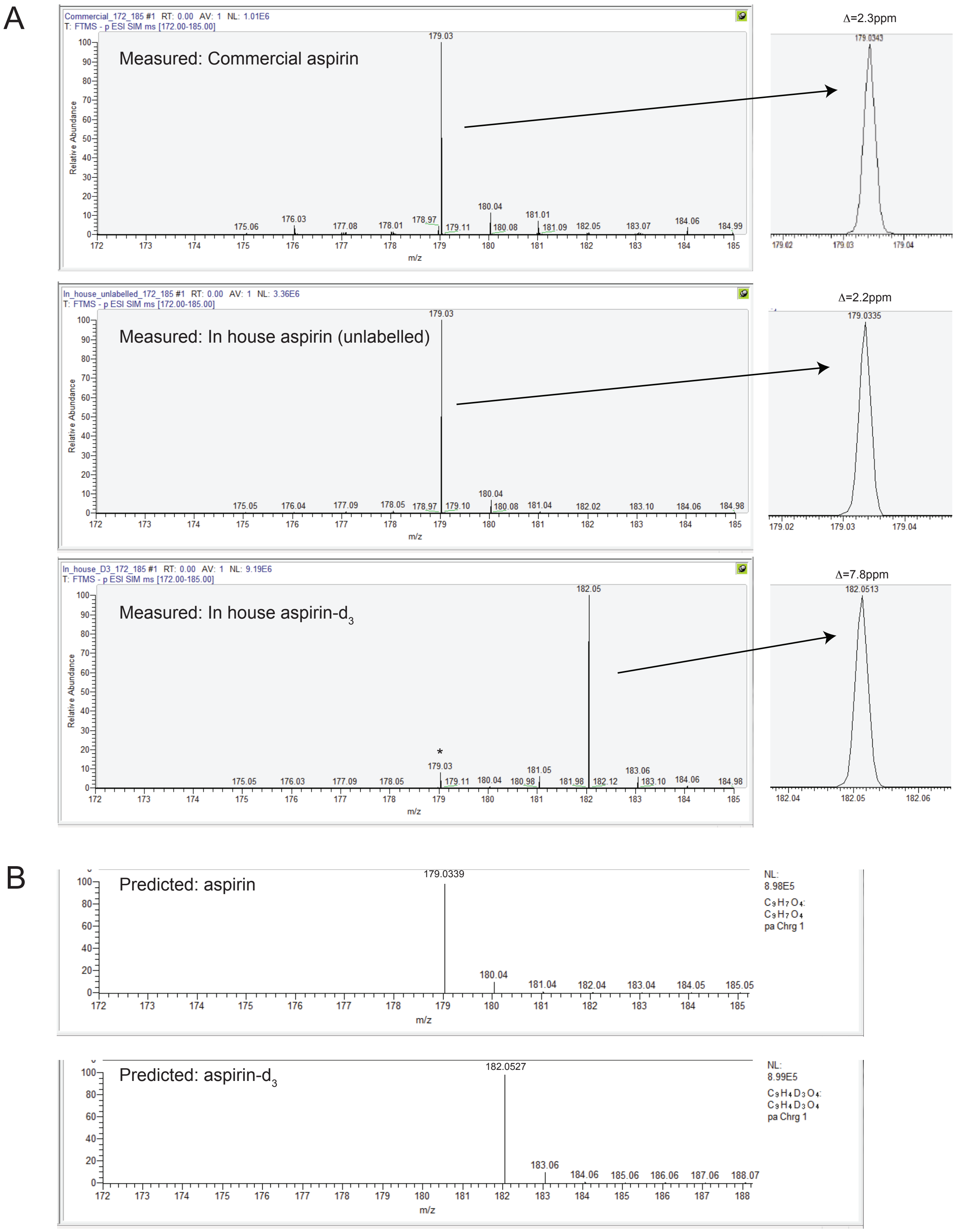


**Figure S3. Confirmation of aspirin-d$_3$ synthesis and discrimination from unlabelled aspirin by mass spectrometry.** A. Full mass spectrum of commercially sourced aspirin (upper), in house synthesised unlabelled aspirin (middle) and in house synthesised aspirin-d$_3$ (bottom). m/z range of 172-185 is shown. Zoomed peak data are shown to the right with difference from predicted mass shown in ppm above each. B. Xcalibur Qual Browser predicted spectra for the proton-dissociated froms of unlabelled aspirin ($C_9H_7O_4$ - upper), and aspirin-d$_3$ ($C_9H_4D_3O_4$ - lower). * Asterisk indicates MS system contamination with unlabelled aspirin rather than sample contamination, as both aspirin and aspirin-d$_3$ peaks were evident in acetonitrile only control runs after this analysis (not shown).

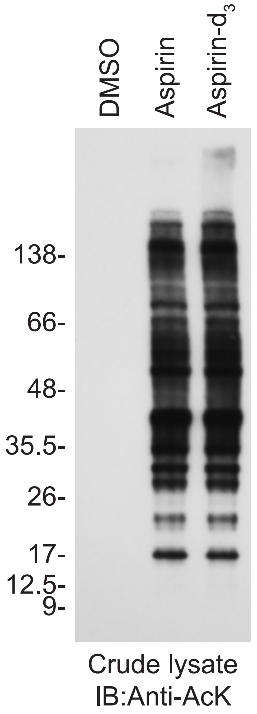


**Figure S4. Confirmation of aspirin-induced acetylation in the large-scale proteomic experiment.** 20 μg of total protein from crude cell lysates from the experiment described in Figure 2A were fractionated by SDS-PAGE and analysed by immunoblot using anti-acetylated lysine antibodies.

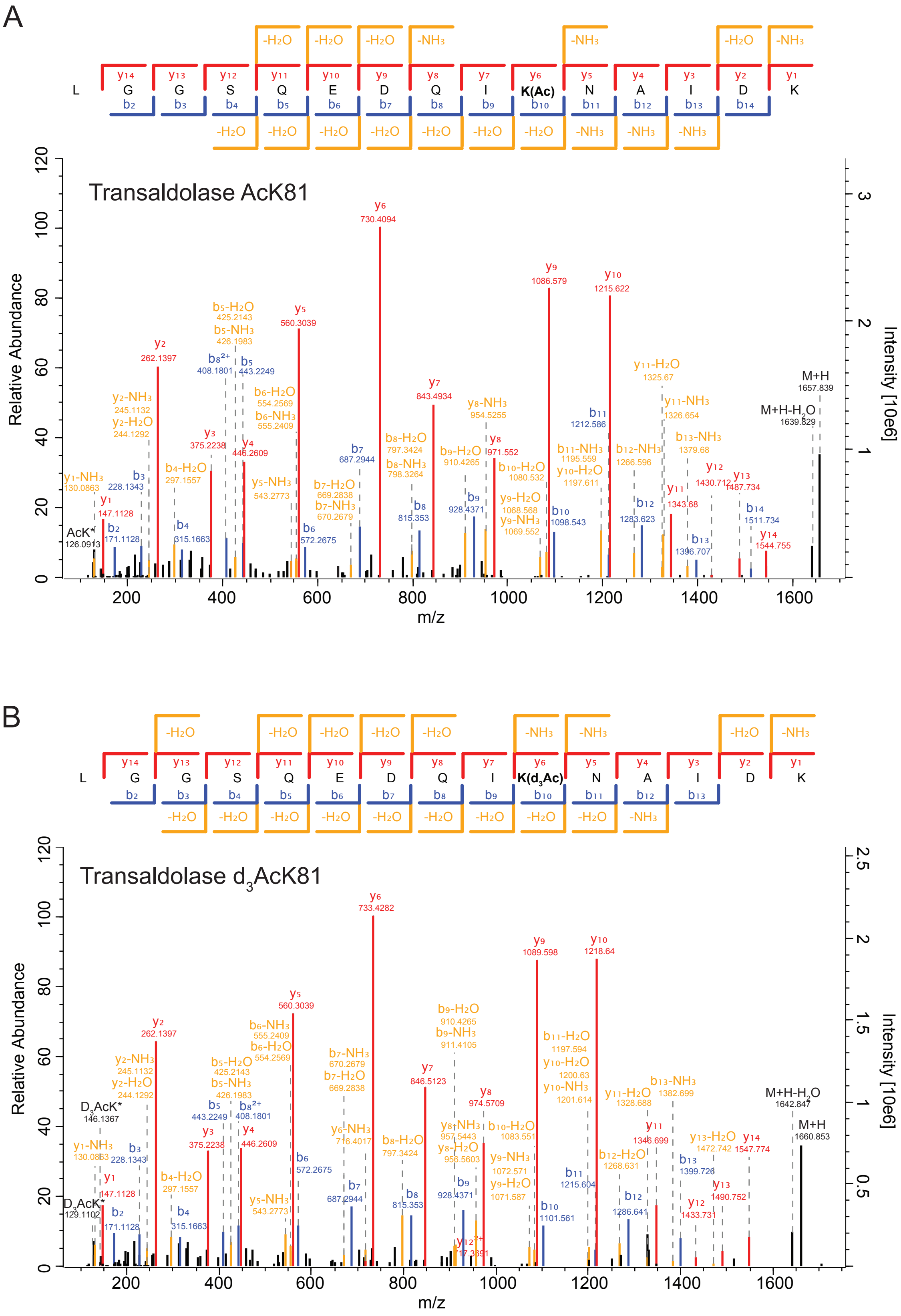


**Figure S5. Example MS/MS spectra of unlabelled acetylation, and aspirin-$d_3$-mediated acetylation.** MS/MS spectra of the peptide from transaldolase showing acetylation at lysine 81 for unlabelled aspirin (A), and aspirin-$d_3$ (B). y and b series ions are shown in red and blue respectively, with ammonia or water loss ions shown in orange. Intact ions (M) and diagnostic peaks (*) are annotated in black.

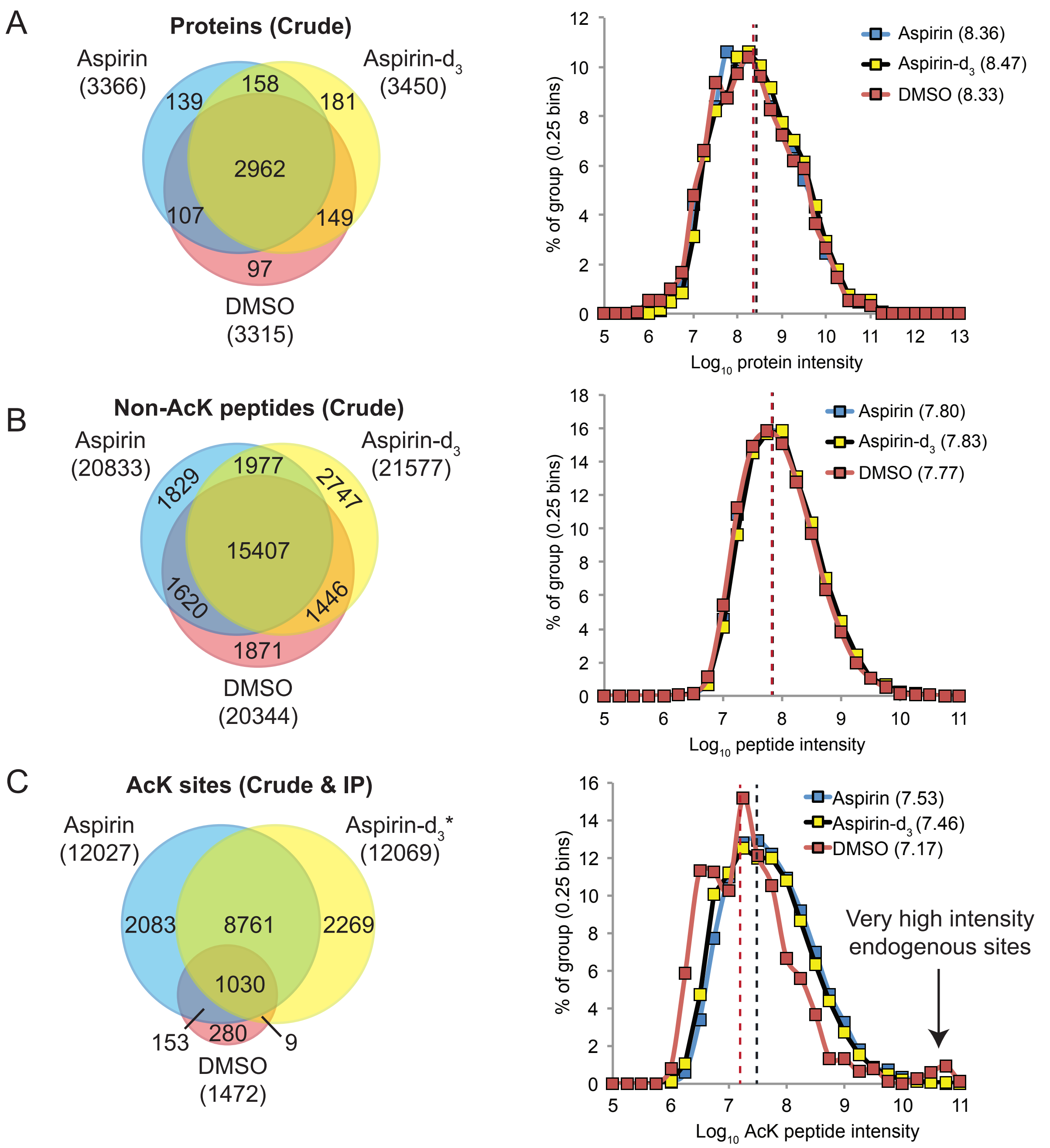


D

| | 95% Confidence interval of the Hodges-Lehmann difference between medians | | |
|---|---|---|---|
| **Data set** | **Aspirin vs Aspirin-d3** | **Aspirin vs DMSO** | **Aspirin-d3 vs DMSO** |
| (A) Protein | 0.020 to 0.111 | -0.098 to -0.004 | -0.163 to -0.070 |
| (B) Non-AcK peptide* | 0.018 to 0.049 | -0.032 to -0.002 | -0.066 to -0.035 |
| (C) AcK peptide | -0.100 to -0.060 | -0.407 to -0.323 | -0.329 to -0.245 |

**Figure S6. Frequency distributions of protein intensity, non-acetylated peptide intensity and acetylated peptide intensity for samples derived from DMSO, aspirin and aspirin-$d_3$ treated cells.** Overlap and frequency distribution of protein intensity (A), non-acetylated peptide intensity (B), and acetylated peptide intensity (C) among the three cell groups for proteins identified. The small group of extremely high intensity acetylated peptides from the DMSO prep are indicated. Median intensities are indicated in parentheses and as broken lines in charts. D. 95% confidence intervals of the differences between the median values of $\log_{10}$ of protein or peptide intensities described in A-C. This is calculated by the Hodges-Lehmann estimation based on the Mann-Whitney test using Graphpad Prism 6.

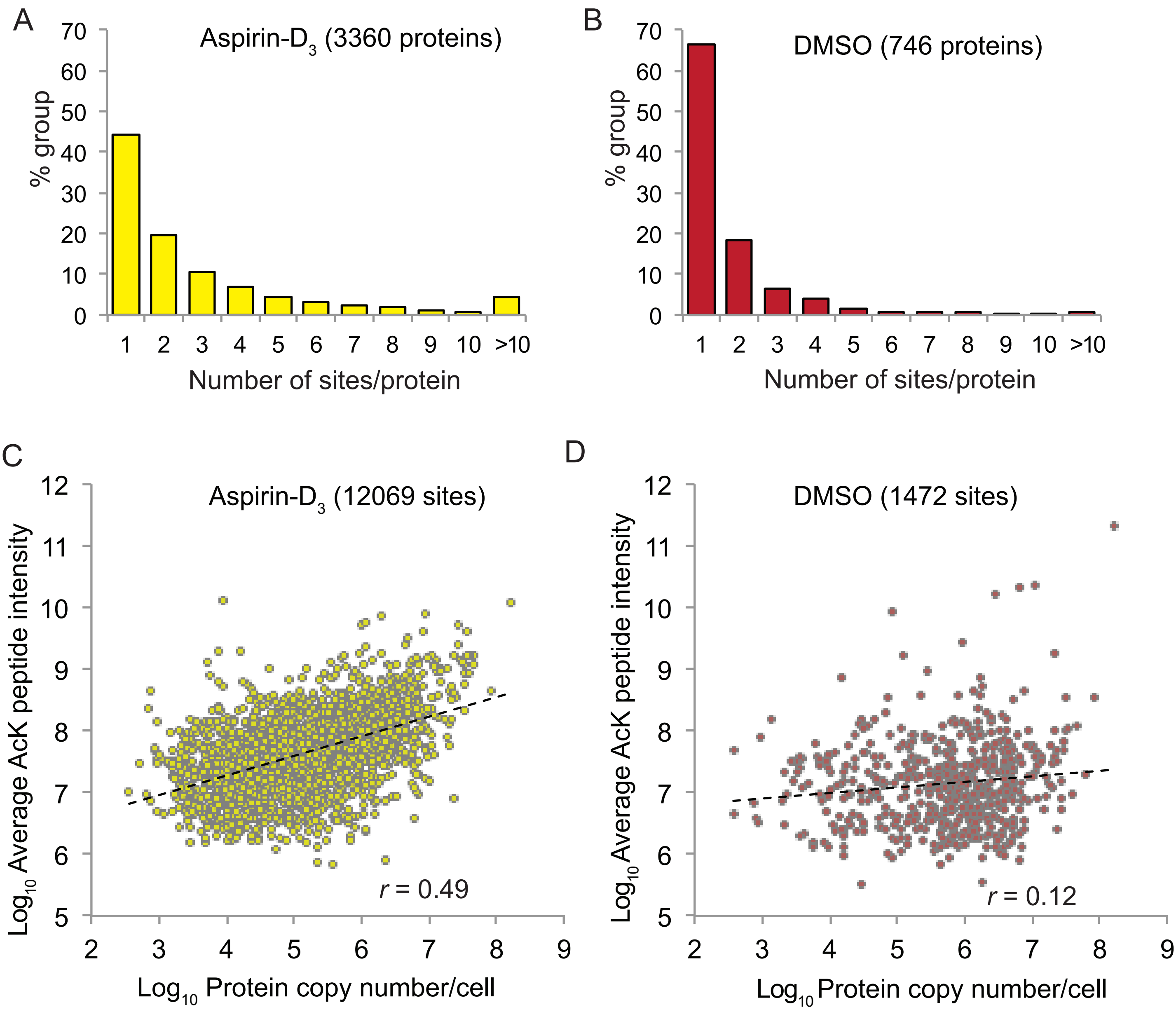


**Figure S7. Acetylated peptide intensity correlates with total protein intensity for aspirin-mediated acetylation, but not for endogenous acetylation.** A and B. Frequency distributions of numbers of $d_3$-AcK sites per protein identified in aspirin-$d_3$-treated cells (A), and AcK sites per protein in DMSO treated cells (B). Scatter plots of $\log_{10}$ protein copy number per cell for acetylated proteins (x-axis) and the $\log_{10}$ average acetylated peptide intensity (y-axis) for aspirin-$d_3$ treated cells (C) and DMSO treated cells (D). Lines of best-fit (broken black) and Pearson correlation coefficients are indicated.

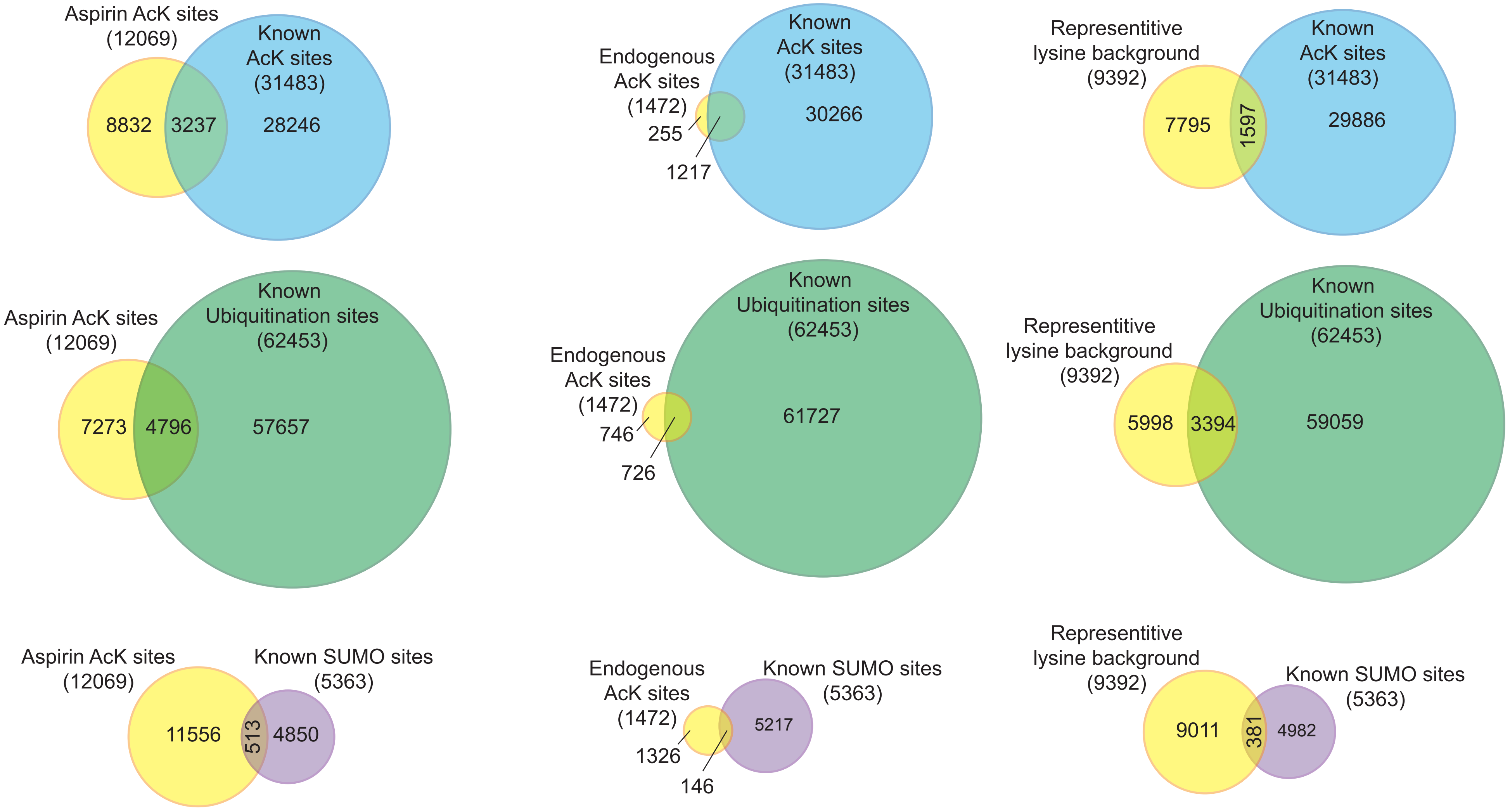


**Figure S8. Overlap between the aspirin-mediated lysine acetylome with known sites of acetylation, ubiquitination and SUMOylation.**

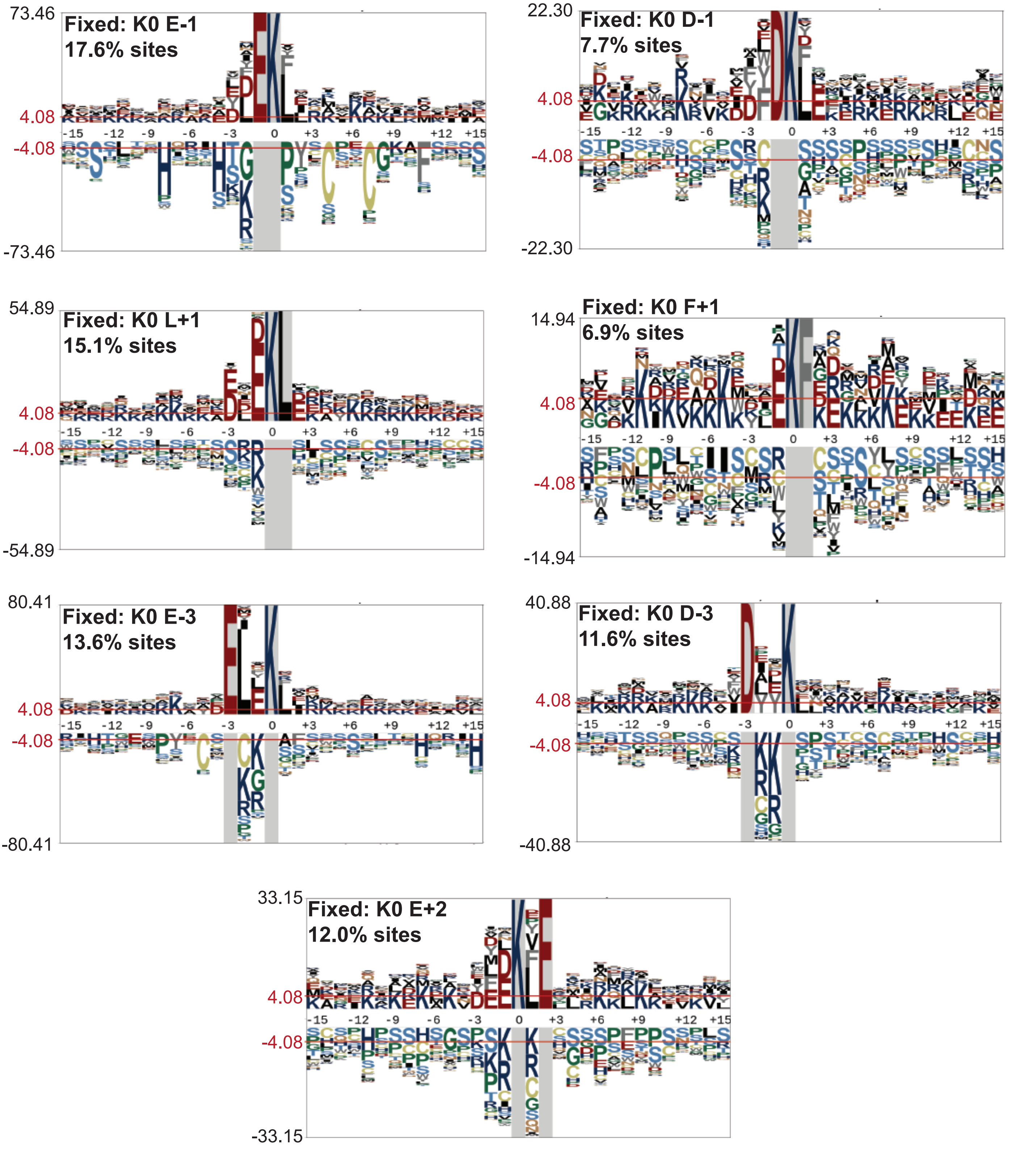


**Figure S9. Lysines acetylated by aspirin tend to exist within acidic sequences.** pLogos (ref - O'Shea JP, Chou MF, Quader SA, Ryan JK, Church GM, & Schwartz D. (2013). Nat Methods 10, 1211-1212.) based upon the 12069 set of aspirin-mediated acetylated lysines with both the target lysine and a second amino acid fixed (as indicated). Note, fixing an acidic amino acid at positions -1, -3 or +2 reduces the frequency of other acidic amino-acids appearing in the logo. Note also the y-axis scale is different for each logo. Y-axis is log-odds of the binomial probability.

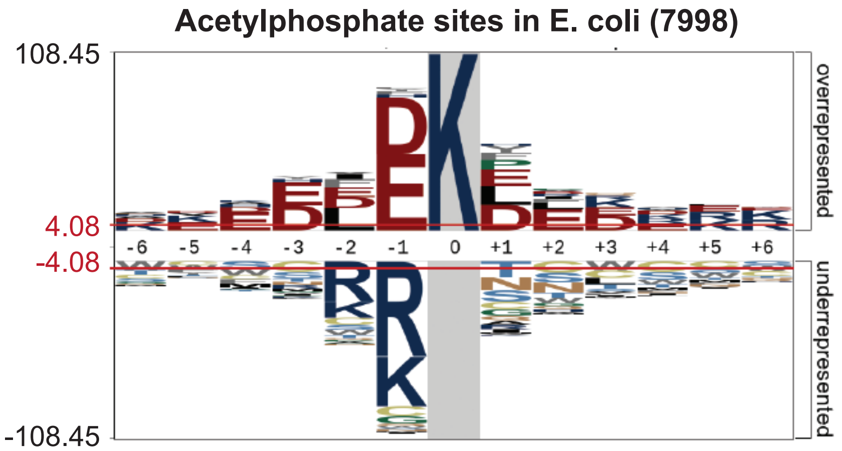


**Figure S10. Sequence logo analysis of 7998 lysines from E. coli thought to be non-enzymatically acetylated by acetylphosphate.** Note the preference for acidic residues close to the target lysine, similar to the non-enzymatic acetylation of proteins by aspirin, Data taken from Weinert, B. T., Iesmantavicius, V., Wagner, S. A., Scholz, C., Gummesson, B., Beli, P., Nystrom, T., and Choudhary, C. (2013) Mol. Cell 51, 265-272).

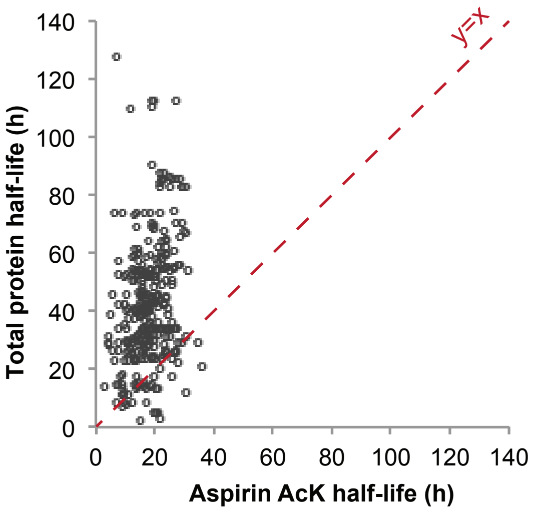


**Figure S11. Influence of protein half-life on aspirin-mediated protein acetylation half-lives.** Relationship between aspirin-mediated lysine acetylation half-life and total protein half-life for 331 sites. y=x line is indicated as described in Boisvert, F. M., Ahmad, Y., Gierlinski, M., Charriere, F., Lamont, D., Scott, M., Barton, G., and Lamond, A. I. (2012) Mol Cell Proteomics 11, M111 011429. In general acetyltion half-lives (median = 21h 17min) are much shorter than protein turnover half-lives (median = 39h 58min), and there is a weak correlation between the two (*r* = 0.26).
